# Whole-Genome Sequencing of a 900-Year-Old Human Skeleton Supports Two Past Migration Events from the Russian Far East to Northern Japan

**DOI:** 10.1093/gbe/evab192

**Published:** 2021-08-19

**Authors:** Takehiro Sato, Noboru Adachi, Ryosuke Kimura, Kazuyoshi Hosomichi, Minoru Yoneda, Hiroki Oota, Atsushi Tajima, Atsushi Toyoda, Hideaki Kanzawa-Kiriyama, Hiromi Matsumae, Kae Koganebuchi, Kentaro K Shimizu, Ken-ichi Shinoda, Tsunehiko Hanihara, Andrzej Weber, Hirofumi Kato, Hajime Ishida

**Affiliations:** 1 Department of Human Biology and Anatomy, Graduate School of Medicine, University of the Ryukyus, Nishihara, Japan; 2 Department of Bioinformatics and Genomics, Graduate School of Medical Sciences, Kanazawa University, Kanazawa, Japan; 3 Department of Legal Medicine, Graduate School of Medicine, University of Yamanashi, Chuo, Japan; 4 The University Museum, The University of Tokyo, Tokyo, Japan; 5 Department of Anatomy, Kitasato University School of Medicine, Sagamihara, Japan; 6 Department of Biological Sciences, Graduate School of Science, The University of Tokyo, Tokyo, Japan; 7 Comparative Genomics Laboratory, National Institute of Genetics, Mishima, Japan; 8 Department of Anthropology, National Museum of Nature and Science, Tsukuba, Japan; 9 Kihara Institute for Biological Research (KIBR), Yokohama City University, Yokohama, Japan; 10 Department of Molecular Life Science, School of Medicine, Tokai University, Isehara, Japan; 11 Department of Biological Structure, Kitasato University Graduate School of Medical Sciences, Sagamihara, Japan; 12 Advanced Medical Research Center, Faculty of Medicine, University of the Ryukyus, Nishihara, Japan; 13 Department of Evolutionary Biology and Environmental Studies, University of Zurich, Zurich, Switzerland; 14 Department of Anthropology, University of Alberta, Edmonton, Alberta, Canada; 15 Research Centre “Baikal Region”, Irkutsk State University, Irkutsk, Russia; 16 Laboratoire Méditerranéen de Préhistoire Europe Afrique (LAMPEA) – UMR 7269, Aix-Marseille Université, Aix-en-Provence, France; 17 Centre for Ainu and Indigenous Studies, Hokkaido University, Sapporo, Japan

**Keywords:** prehistoric Okhotsk people, ancient genomics, next-generation sequencing, population history, admixture dating

## Abstract

Recent studies on paleogenomics have reported some Paleolithic and Neolithic genomes that have provided new insights into the human population history in East and Northeast Asia. However, there remain some cases where more recent migration events need to be examined to elucidate the detailed formation process of local populations. Although the area around northern Japan is one of the regions archaeologically suggested to have been affected by migration waves after the Neolithic period, the genetic source of these migrations are still unclear. Thus, genomic data from such past migrant populations would be highly informative to clarify the detailed formation process of local populations in this region. Here, we report the genome sequence of a 900-year-old adult female (NAT002) belonging to the prehistoric Okhotsk people, who have been considered to be the past migrants to northern Japan after the Neolithic period. We found a close relationship between NAT002 and modern Lower Amur populations and past admixture events between the Amur, Jomon, and Kamchatka ancestries. The admixture dating suggested migration of Amur-related ancestry at approximately 1,600 BP, which is compatible with the archaeological evidence regarding the settlement of the Okhotsk people. Our results also imply migration of Kamchatka-related ancestry at approximately 2,000 BP. In addition, human leukocyte antigen (HLA) typing detected the HLA-B*40 allele, which is reported to increase the risk of arthritis, suggesting the genetic vulnerability of NAT002 to hyperostosis, which was observed around her chest clavicle.

**Significance**

Whole-genome sequence data of a 900-year-old human skeleton excavated from the northern part of the Japanese archipelago indicated genetic affinity with populations in the Russian Far East, including the Amur Basin and the Kamchatka Peninsula, and a Neolithic Jomon individual, an indigenous hunter–gatherer in the Japanese archipelago. Population genetic analyses support two past admixture events between the ancient human populations in the border region between the Japanese archipelago and Russian Far East.

## Introduction

**Fig. 1.**
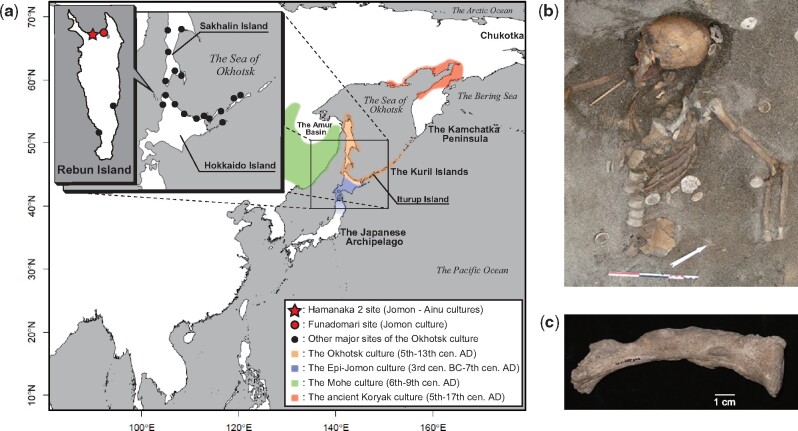
(*a*) Geographic locations of the Hamanaka 2 site from which NAT002 was excavated, the Funadomari site from which F23 was excavated, and other archaeological sites of the Okhotsk culture, and geographic ranges of the Okhotsk culture and neighboring cultures. (*b*) Skeleton of the adult female of the prehistoric Okhotsk culture (NAT002) from the Hamanaka 2 site. (*c*) The left clavicle of NAT002. Hyperostosis can be observed on the surface.

Paleogenomics is a powerful tool that allows snapshots of genetic features to be taken for past human populations. Recent ancient genomic studies have reported many Asian Paleolithic and Neolithic human genomes and provided new insights into the process of human colonization in East Eurasia (Fu et al. 2013; Raghavan et al. 2014; Siska et al. 2017; Lipson et al. 2018; McColl et al. 2018; Sikora et al. 2019; Yang et al. 2020). Especially, Tianyuan (Fu et al. 2013), MA1 (Raghavan et al. 2014), and Yana_UP (Sikora et al. 2019) are key ancient genomes for understanding the early human population history in East Eurasia. In a phylogenetic tree, Tianyuan forms a cluster with Papuan and East Eurasian populations, whereas MA1 and Yana_UP are genetically close to West Eurasian populations. Although the detailed routes are still contentious, these genomes suggest the following two past migration waves to East Eurasia: the southern wave, which seems to run along the southern coastal region of the Eurasian continent, and the northern wave, which probably runs through the Siberian and Eurasian steppe regions. The southern migration wave seems to have diversified into the local populations in East Asia (defined in this paper as a region including China, Japan, Korea, Mongol, and Taiwan) and Southeast Asia. The northern migration wave mixed with the southern wave, probably in Siberia, and became the origin of the Chukot–Kamchatka (the Chukotka and Kamchatka Peninsula, see fig. 1*a*) populations and Native Americans (Raghavan et al. 2014). Moreover, subsequent several studies (de Barros Damgaard, Marchi, et al. 2018; de Barros Damgaard, Martiniano, et al. 2018; Jeong et al. 2018, 2020; Ning et al. 2020) that have analyzed ancient genomes since the Neolithic period and Bronze Age, have revealed the population dynamics after the Neolithic period on the Asian continent.

Regarding the Japanese archipelago, the genomes of several individuals of the Jomon people (Kanzawa-Kiriyama et al. 2017, 2019; Gakuhari et al. 2020), who are the Neolithic indigenous population in this region, have been reported, which suggests their deep divergence from the southern wave lineage. However, the genetic history of the Japanese populations after the Neolithic period is still unclear because of the lack of ancient genomic data of that period. Some previous studies (Kanzawa-Kiriyama et al. 2017, 2019; Gakuhari et al. 2020) suggested that modern Japanese populations (i.e., the Ainu, mainland Japanese, and Ryukyuan) were affected by gene flows from populations in continental East and/or Northeast Asian populations (in this paper, Northeast Asia corresponds to the Russian Far East) after the Neolithic period, although the detailed sources and processes of these migrations remain unclear. Therefore, revealing the genetic features of such past migrants is also important for gaining a better understanding of the population history around the Japanese archipelago. As suggested by archaeological evidence, one of the past major migration events that seems to have occurred after the Neolithic period in these regions is the settlement in northern Japan by the prehistoric Okhotsk people.

The Okhotsk culture, a prehistoric hunting and gathering culture with advanced ocean-based fishing and hunting technologies, was distributed around the southern coastal regions of the Sea of Okhotsk (fig. 1*a*) from the fifth to thirteenth centuries (Befu and Chard 1964; Vasiliefsky 1978). The dependence on marine resources, which is the most characteristic feature of the Okhotsk culture, was revealed by previous zooarchaeological and isotopic studies (Hudson 2004; Naito et al. 2010). In fact, the distribution of archaeological sites of the Okhotsk culture is apparently restricted to the coastal area (fig. 1*a*). This feature is remarkably different from the indigenous Jomon (14,000 to 300 BC in northern Japan) and subsequent Epi-Jomon (third century BC to seventh century AD, fig. 1*a*) and Satsumon (eighth to fourteenth centuries AD) cultures in the Japanese archipelago. In terms of the relationship between the Okhotsk culture and the neighboring ancient cultures in Northeast Asia, some of the potteries, iron wares, and bronze wares excavated from archaeological sites of the Okhotsk culture are similar to those discovered from sites of the Mohe culture (sixth to ninth centuries AD, fig. 1*a*), which developed in the Primorski region (Kikuchi 1995). In addition, statues and fishhooks made of walrus tusks excavated from archaeological sites of the Okhotsk culture suggest interaction between the Okhotsk culture and the ancient Koryak culture (fifth to seventeenth centuries AD, fig. 1*a*) because walrus are currently distributed only in the Arctic Ocean and the Bering Sea (Kikuchi 2004). However, whether these transcultural commonalities of archaeological relics were derived from human migration or from trade without migration, remains unclear. The origin of the Okhotsk people has long been discussed by archaeologists and anthropologists. Morphological studies of the skeletal remains of the Okhotsk people have suggested their similarity to populations currently living around the Amur Basin and in northern Sakhalin (Ishida 1988, 1996; Komesu et al. 2008). In addition, the results of mitochondrial DNA analysis support the morphological evidence. Mitochondrial DNA haplogroups Y1, G1b, and N9b, which were shared among the Lower Amur populations at high frequencies, were commonly detected from Okhotsk skeletal remains (Sato et al. 2009b), which suggests that the Okhotsk people originated in the Lower Amur region; however, because of a lack of comprehensive genome-wide data of the Okhotsk people, a final conclusion has yet to be reached. Therefore, the primary motivation behind this study was to understand the genetic origins of the Okhotsk people based on genome-wide data analyses.

Based on archaeological evidence, the Okhotsk culture disappeared around the thirteenth century, the cause of which is also still unclear. In connection with this, the relationship between the Okhotsk and subsequent Ainu cultures has been investigated (Yamaguchi 1981; Utagawa 2002; Komesu et al. 2008; Sato et al. 2009b; Adachi et al. 2018). Archaeologists have considered that bear worship, which is a religious practice widely observed among the northern Eurasian ethnic groups, including the Ainu, Finns, Nivkh, and Sami, was also shared by the Okhotsk people. On the other hand, no traces of such a religious practice have ever been discovered from archaeological sites of the Jomon and Epi-Jomon periods, which were anterior to the Ainu cultural period. Bear worship in the Ainu culture is seen in *iomante*, a ceremony in which a bear is sacrificed and its spirit is sent to the world of *kamuys* (a *kamuy* is a divine being in the Ainu culture). For the Okhotsk culture, the enshrined skulls of brown bears have been discovered in some dwelling sites, suggesting a religious custom associated with bear worship. This implies that the Okhotsk culture contributed to the forming of the Ainu culture (Utagawa 2002). Some craniological and mitochondrial DNA studies have implied genetic affinity between the Ainu and Northeast Asian populations. Statistical analyses based on cranial measurements suggest that Northeast Asians had a slight genetic influence on the Ainu settling along the Okhotsk Sea coast (Yamaguchi 1981). Mitochondrial and Y chromosomal DNA analyses of the modern Ainu indicate similarities in the maternal and paternal gene pools between the Nivkh and Ainu (Tajima et al. 2004). These findings have been interpreted as potential gene flow from Northeast Asia to northern Japan. Moreover, ancient mtDNA and cranial nonmetric variation studies have suggested close relationships between the Okhotsk people and the Lower Amur populations, as well as gene flow from the Lower Amur populations to the Ainu intermediated by the Okhotsk people (Komesu et al. 2008; Sato et al. 2009b; Adachi et al. 2018), which supports the archaeological hypothesis regarding the contribution of the Okhotsk people to the establishment of the Ainu culture. However, these studies are based on a small number of morphological or genetic markers and cannot provide a final conclusion on the genetic contribution of the Okhotsk people to the Ainu because such studies cannot statistically assess the population admixture. By contrast, a recent genome-wide single nucleotide polymorphism (SNP) analysis of modern individuals, which can statistically evaluate population admixture, did not detect gene flow from the Lower Amur populations to the Ainu, although this would be expected if the Okhotsk people had made a genetic contribution to the Ainu (Jeong et al. 2016). Therefore, although a number of previous studies using various approaches have been conducted with the aim of elucidating the formation process of the Ainu population, whether the Okhotsk people made a genetic contribution to this process remains unclear. Thus, the second goal of this study was to address this issue based on ancient genome-wide data.

To investigate the formation processes of the Okhotsk people and their genetic and cultural contributions to the Ainu, an international multidisciplinary team embarked on an excavation of the Hamanaka 2 site on Rebun Island, the northernmost part of the Japanese Archipelago (fig. 1*a*). In 2013, the well-preserved skeletal remains of an adult female (NAT002) with severe hyperostosis were recovered from the upper surface of a shell mound corresponding to the final stage of the Okhotsk period (fig. 1*b and c*). The radiocarbon (^14^C) calibrated age of NAT002 was estimated to be 1,060–1,155 calAD (68.2%), and hyperostosis was diagnosed as synovitis, acne, pustulosis, hyperostosis, osteitis (SAPHO) syndrome without dermatological observations (Okamoto et al. 2016). In this study, we performed whole-genome sequencing of NAT002 to resolve the issues regarding the origins of the Okhotsk people and their genetic contribution to the Ainu population. We compared the NAT002 genome with modern East and Northeast Asian populations and recently reported ancient Northeast Asians such as Devil’s Gate individuals, Neolithic human skeletal remains excavated from the Primorsky region (Siska et al. 2017; Sikora et al. 2019) and F23, a Neolithic Jomon individual recovered from the Funadomari site (fig. 1*a*) on Rebun Island (Kanzawa-Kiriyama et al. 2019). We also attempted to identify the genetic background of SAPHO syndrome-like hyperostosis observed in some bones of NAT002.

## Results and Discussion

### Basic Statistics and DNA Authenticity of NAT002 Sequence Data

We obtained two DNA extracts from two tooth samples processed in two distinct genetic laboratories (see Materials and Methods). Surprisingly, the DNA length distributions for the two obtained extracts, especially extract 2, were unusually long as ancient DNA molecules (supplementary fig. 1, Supplementary Material online). Although these DNA length distributions made us suspect modern DNA contamination, the same mtDNA sequence and autosomal short tandem repeat (STR) profile were obtained from the two extracts by preliminary Sanger sequencing and STR fragment analysis. Therefore, we attempted to perform next-generation sequencing (NGS) on both extracts. Because of a lack of short DNA molecules in extract 2, artificial DNA shearing was performed to prepare Illumina platform libraries from the extract, although this is a nonstandard procedure for ancient DNA analysis (see Materials and Methods).

As a result of shotgun sequencing and subsequent quality control, approximately 4 billion sequence reads were obtained from 18 Illumina platform libraries. Mapping rates to the reference genome (hs37d5) were substantially high for all of the libraries derived from the two independent extracts (67.7% average for extract 1 and 72.2% average for extract 2). As described in supplementary text S1, Supplementary Material online, we finally concluded that the majority of the DNA molecules in both extracts was derived from the authentic NAT002 DNA based on a high proportion of identity-by-descent (IBD) between extracts (π^=0.9308), the observed deamination and depurination patterns (supplementary figs. S2 and S3, Supplementary Material online), the estimated sufficiently low modern DNA contamination rates (supplementary figs. S4–S6, Supplementary Material online), the consistent results of preliminary population genetic analyses between read fractions based on insert length or PMD score (supplementary figs. S7 and S8, Supplementary Material online), and the sampling situation (see Sampling, DNA extraction, Library Build, and Sequencing in the Materials and Methods section). We speculated that the miraculous survival of the long DNA molecules could be attributed to the burial environments, cold climates, relatively recent age of NAT002, and immediate preservation in a freezer, as also discussed in supplementary text S1, Supplementary Material online.

The total numbers of sequence reads mapped to the reference genome after merging the alignment files (BAM files) derived from the distinct extracts are summarized in supplementary table S1, Supplementary Material online. The ratio of reads mapped to Y and X chromosomes, the *R*_Y_ value, was 0.0017, which was calculated using ry_compute.py (Skoglund et al. 2013), indicating that NAT002 was female. This finding was consistent with previous results regarding morphological- and Amelogenin gene-based sex identification (Okamoto et al. 2016). The total error rate of the NAT002 sequence estimated based on chimpanzee (PanTro2) and high-quality modern Japanese genomes was 0.11%, indicating a relatively low level of postmortem damage (supplementary fig. S2*c*, Supplementary Material online). We re-estimated modern DNA contamination rates for the full NAT002 data. For instance, the contamination rates based on mtDNA and chromosome 1 were 1% (supplementary fig. S9, Supplementary Material online) and 0.055% (supplementary fig. S10, Supplementary Material online), respectively. Posterior distributions of contamination rates based on the other autosomes, which suggest the sufficient low contamination rates, are also shown in supplementary figure S10, Supplementary Material online.

Finally, we obtained the NAT002 genome sequence with an average depth of 35.03× (supplementary fig. S11, Supplementary Material online). We applied the method of the Simons Genome Diversity Project (SGDP) (Mallick et al. 2016) for genotype calling and depth-based filtering to the NAT002 sequence data using cmakefilter in cTools. The divergence between NAT002 and chimpanzee was slightly higher than that between chimpanzee and the modern Japanese sequence with 30-fold coverage (supplementary fig. S12, Supplementary Material online), probably because of postmortem deamination. We also applied these calling and filtering procedures to two individuals of the modern Nivkh (Raghavan et al. 2014) and F23 (Kanzawa-Kiriyama et al. 2019), which is the high-coverage genome of the 3,500-year-old Neolithic Jomon individual from the Funadomari site (fig. 1*a*) located on Rebun Island, for comparison with NAT002.

### Genetic Relationships between NAT002 and the Neighboring Populations

**Fig. 2.**
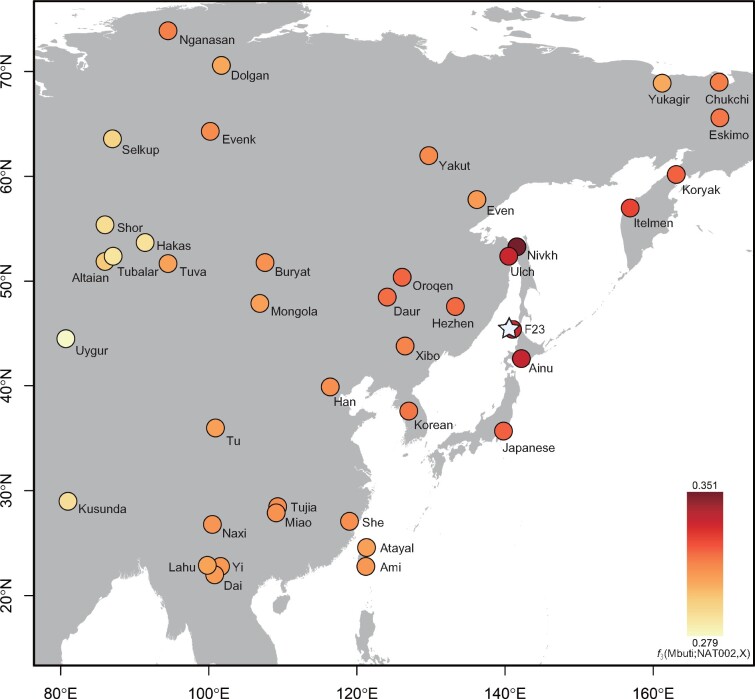
The result of the outgroup *f*_3_ test. *f*_3_(Mbuti; NAT002, X) was calculated (modern Asian populations and F23 were used as population X). The star indicates the location of the site from which NAT002 was excavated.

**Fig. 3.**
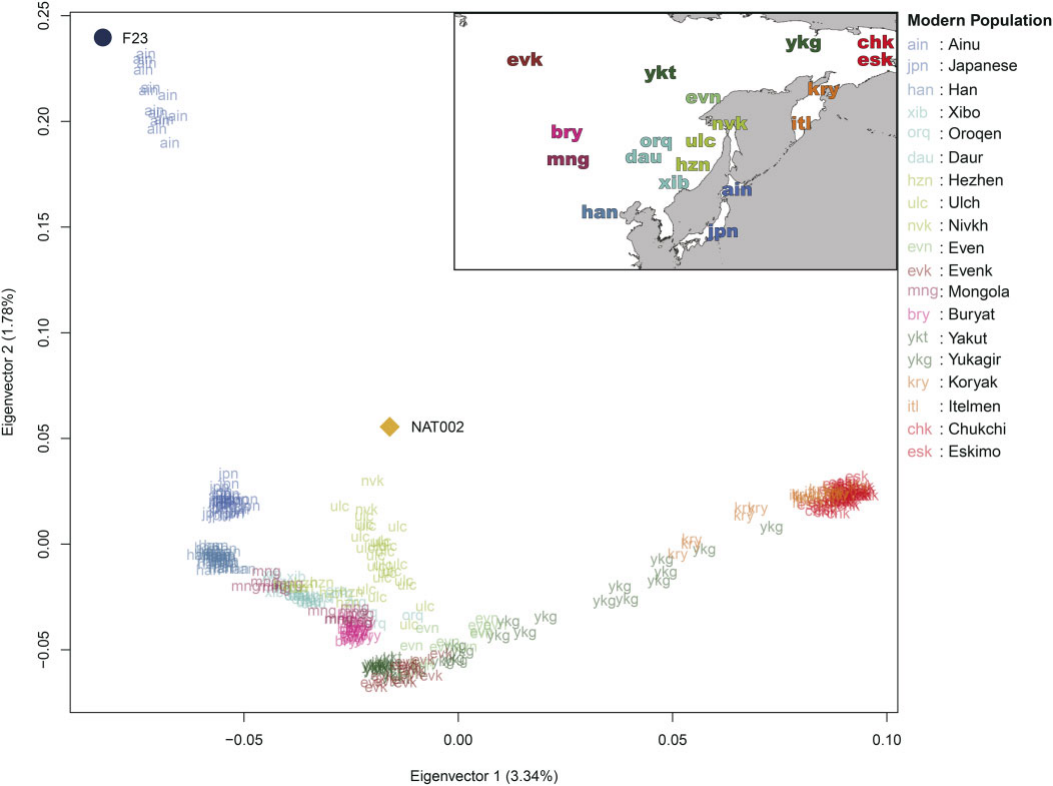
PCA plot using NAT002, F23, and modern East and Northeast Asian populations. The contribution rate of each eigenvector is indicated in parentheses.

To assess the genetic affinity of NAT002 to Northeast Asian human populations, outgroup *f*_3_-statistics were calculated based on 49,079 SNPs. The *f*_3_(Mbuti; NAT002, X) indicated that NAT002 was closely related to the Nivkh, Ainu, Ulch, and F23 located in the neighboring regions, followed by the Kamchatka populations, including the Itelmen and Koryak, mainland Japanese, and Upper Amur populations such as the Oroqen, Daur, and Hezhen (fig. 2). A principal component analysis (PCA) plot based on 49,079 SNPs also indicated that NAT002 was genetically close to the Nivkh and Ulch (fig. 3), supporting the findings of previous morphological and mitochondrial DNA studies (Ishida 1988, 1996; Komesu et al. 2008; Sato et al. 2009b).

**Fig. 4.**
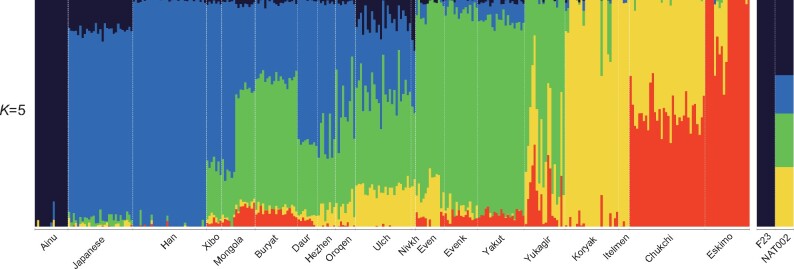
Stacked bar plots for the results of ADMIXTURE analysis when *K *=* *5, which showed the minimum cross-validation error.

**Fig. 5.**
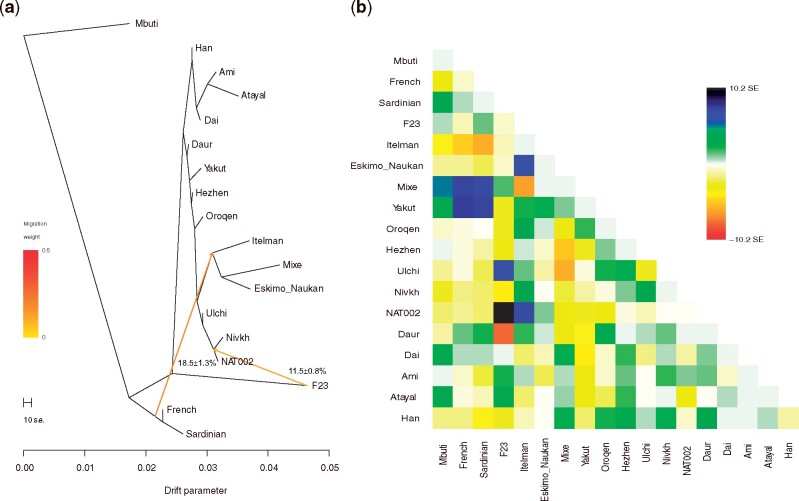
(*a*) Phylogenetic tree and (*b*) residual matrix constructed using TreeMix when two migration events were assumed.

We also ran ADMIXTURE (Alexander et al. 2009) from *K *=* *2 to *K* = 10. The minimum cross-validation (CV) error was observed when *K *=* *5 (supplementary fig. S13, Supplementary Material online). NAT002 was represented as an admixed individual among the four genetic components (fig. 4). Green component (24.6%) was widely shared among the Northeastern Asian populations. Blue component (15.8%) was widely shared among the East Asians. Navy component (33.2%) was observed in F23 and the Ainu at a rate of almost 100%. Yellow component (26.4%) was shared by the Itelmen and Koryak at the highest ratio. The component proportion of NAT002 was similar to those of the Lower Amur populations, including the Nivkh and Ulch. Next, to infer the phylogenetic relationships between NAT002 and Northeast Asians, we ran TreeMix (Pickrell and Pritchard 2012) based on 2,556,493 variants observed among NAT002, F23, and the SGDP samples. NAT002, Nivkh, and Ulch formed a monophyletic cluster (fig. 5*a*), corresponding to the results of the PCA and ADMIXTURE. When two migration events were assumed, gene flow was detected from F23 to the common ancestor between NAT002 and Nivkh (fig. 5*a*). Large residuals were also observed between F23 and the Ulch, and between NAT002 and the Itelman, suggesting gene flow between them (fig. 5*b*). The genetic affinity between the Ulch and Jomon/Ainu was previously reported by Jeong et al. (2016) and Kanzawa-Kiriyama et al. (2019). In addition, we investigated the genetic affinity between NAT002 and Devil’s Gate individuals, the early Neolithic East Asians (Siska et al. 2017; Sikora et al. 2019), by calculating outgroup *f*_3_(Mbuti; DevilsGate, X). Among the tested populations, NAT002 showed the second highest *f*_3_ value after the Nivkh (supplementary fig. S14, Supplementary Material online). Although this result might have been affected by the shared bias among the ancient genomes, such an overestimate of the genetic affinity between ancient genomes is likely to be mainly a result of reference bias caused by the read shortness, high frequency of postmortem damage, and insufficient depth in the two ancient genomes compared (Günther and Nettelblad 2019). The degree of reference bias in the Devil’s Gate genomes would be relatively strong because of the low coverage. However, the reference bias in NAT002 genome was not much stronger than the 30-fold coverage of the modern Japanese genome at filtering levels 0 and 1, which were used as genotype call thresholds in this study (supplementary fig. S12, Supplementary Material online). Therefore, the observed affinity between Devil’s Gate individuals and NAT002 was not likely to be strongly overestimated. The high value of *f*_3_(Mbuti; DevilsGate, NAT002) supports the hypothesis that the Okhotsk people originated around the Amur Basin. On the other hand, F23 showed a low *f*_3_ value, suggesting that the migration of the Jomon lineage from northern Japan to the Amur Basin mainly occurred after the Neolithic period, supporting the previous admixture dating in the Ulch (Jeong et al. 2016).

**Fig. 6.**
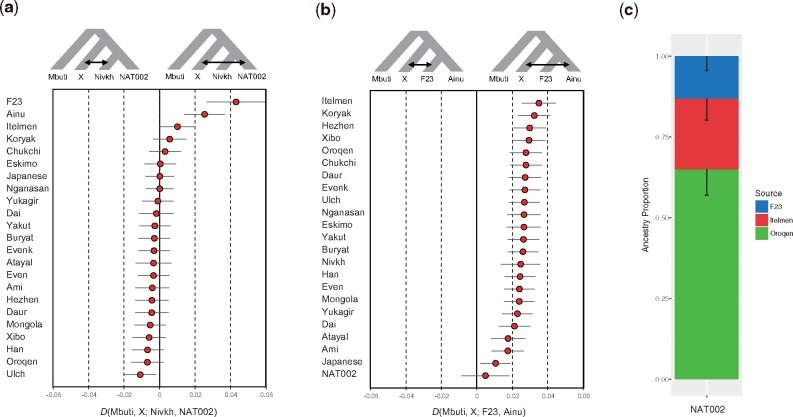
D-statistics and qpAdm model. (*a*) *D*(Mbuti, X; Nivkh, NAT002) and (*b*) *D*(Mbuti, X; F23, Ainu). Error bars indicate two SEs. (*c*) Admixture modeling of NAT002 using qpAdm. The Oroqen, Itelmen, and F23 were used as source populations. Error bars indicate 1 SE.

Considering the topology of the phylogenetic tree (fig. 5*a*), the affinity between the populations in the Amur Basin and NAT002 shown by the *f*_3_(Mbuti; NAT002, X), could be interpreted as a close phylogenetic relationship. On the other hand, the affinities between F23 or Itelmen and NAT002 might be attributed to admixture events after population divergence. To validate the these implied admixture events, we tested *D*(Mbuti, X; Nivkh, NAT002) based on 49,079 SNPs. F23, Ainu, and Itelmen showed significant positive values (fig. 6*a*). The possibility of overestimation of the signal observed in *D*(Mbuti, F23; Nivkh, NAT002) also needs to be considered because of the shared bias between the ancient genomes; however, the reference biases of F23 and NAT002 are not so strong compared with the 30× modern Japanese genome, so the degree of overestimation would not be very serious. Admixture signals were also observed in the test of *D*(Mbuti, F23; Amur, NAT002) and *D*(Mbuti, Itelmen; Amur, NAT002) (supplementary fig. S15, Supplementary Material online). Here, the Amur is a union of the Ulch, Oroqen, Hezhen, Daur, and Xibo. These findings suggest that NAT002 was an admixed individual among three ancestral lineages (the Amur, Chukot–Kamchatka, and Jomon). Next we tested this hypothesis for the past admixture events related to NAT002 using the qpAdm model (Haak et al. 2015). The Oroqen, Itelmen, and F23 were used as representatives of the Amur, Chukot–Kamchatka, and Jomon source populations, respectively. *P* value for the likelihood ratio test (LRT) for the proposed admixture model against full rank model was not significant (*P *=* *0.14), indicating that the NAT002 genome can be explained as a mixture of the Amur, Chukot-Kamchatka, and Jomon ancestries. The estimated ancestry proportions of the Amur, Chukot-Kamchatka, and Jomon were 64.9 ± 8.0%, 21.9 ± 6.4%, and 13.2 ± 4.3%, respectively (fig. 6*c*). Relatively large SEs for proportions of the Amur and Chukot–Kamchataka ancestries might be attributed to the close relationship between their source populations. In fact, yellow and red components observed in the ADMIXTURE analysis (fig. 4), which are predominant among Chukot–Kamchatka populations (the Itelmen, Koryak, Eskimo, and Chukchi), are also broadly observed in the Amur populations such as the Nivkh, Ulch, Oroqen, Hezhen, Daur, and Xibo. This sharing of components seems to be a result of admixture or partially shared genetic drift between the Amur and Chukot–Kamchatka populations. In addition, LRT for the nested models indicated that any two-way admixture models cannot adequately explain the NAT002 genome (*P *=* *7.4 × 10^−4^ for F23-Oroqen, *P *=* *3.9 × 10^−15^ for F23-Itelmen, *P *=* *0.0021 for Itelmen-Oroqen).

### Inferring Local Ancestry and Admixture Dating

**Fig. 7.**
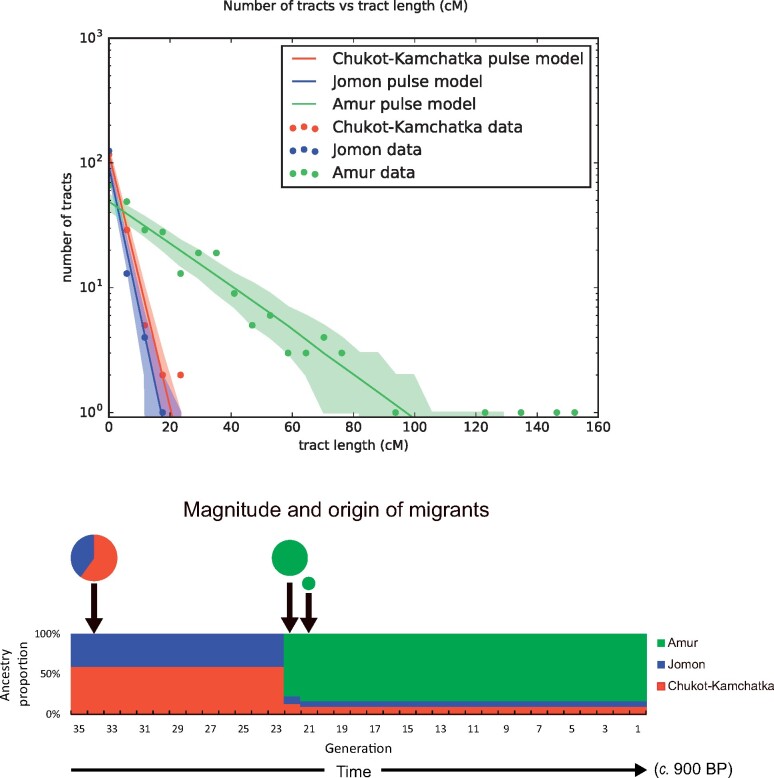
Migration model inferred from the tract length distributions. The single pulse model of three populations was assumed. 0 generation corresponds to the ^14^C age of NAT002 (∼900 BP).

To determine the order of the admixture events among the Amur, Jomon, and Chukot–Kamchatka lineages, we inferred the local ancestries of the NAT002 genome. Although our local ancestry estimation was performed on an intracontinental scale (i.e., all reference populations belonging to East and Northeast Asia), the major parts of the NAT002 genome were successfully assigned to one of the three ancestral haplotypes with posterior probabilities higher than 0.9 (supplementary fig. S16, Supplementary Material online). The proportions of the global ancestries calculated from the local ancestry proportions were as follows: 83.6% of the Amur, 10.0% of the Chukot–Kamchatka, and 6.4% of the Jomon. Considering the ADMIXTURE and qpAdm results (figs. 4 and 6*c*), the proportions of the Jomon and Chukot–Kamchatka ancestries seemed to be underestimated, probably because of the small sample sizes of the Jomon and Kamchatka individuals in the reference populations (only F23 was included in the Jomon reference, and only one Itelman individual was included as representative of the Kamchatka populations in the Chukot–Kamchatka reference, together with five individuals from Chukotka populations such as Chukchi and Eskimo). We assumed the simple pulse migration model to infer the admixture dates among the three lineages. The migration date of the Amur lineage was estimated to be 22 generations before NAT002 (fig. 7). Assuming 30 years per generation and considering the ^14^C age of NAT002 (∼900 BP), migration of Amur-related ancestry occurred approximately 1,600 BP. This corresponds precisely to the beginning of the Okhotsk culture, as indicated by previous archaeological evidence (Befu and Chard 1964; Vasiliefsky 1978); however, our admixture dating was based on ancestry tract length in only one individual. The admixture between the Chukot–Kamchatka and Jomon lineages was estimated to be 35 generations before NAT002 (∼1,950 BP), corresponding to the Epi-Jomon period (third century BC to seventh century AD), which was subsequent to the Jomon period. Although to our knowledge, no archaeological evidence demonstrating human migration from Kamchatka to northern Japan in that period has been presented, a previous ancient DNA study (Adachi et al. 2011) reported an alteration of mitochondrial DNA haplogroup profiles between the Jomon and Epi-Jomon specimens. Haplogroup G1b, which is observed at extremely high frequencies in Kamchatka populations (Starikovskaya et al. 2005), has been detected in 15.0% of Epi-Jomon, but not Jomon specimens. In addition, a recent study reported genetic affinity between an Epi-Jomon specimen from the Tankovoye 1 site on Iturup Island, which is located in the southwestern part of the Kuril Islands (fig. 1*a*), and modern Kamchatka populations, including the Itelmen and Koryak (Moiseyev et al. 2019). These findings support a migration wave from the Kamchatka Peninsula to northern Japan via the Kuril Islands during the Epi-Jomon period. We performed similar admixture dating by adding ten of the unadmixed Ainu individuals to the Jomon reference. In this case, the proportion of the Jomon ancestry in the NAT002 genome increased (64.4% of the Amur, 9.8% of the Chukot–Kamchatka, and 25.8% of the Jomon; supplementary fig. S17, Supplementary Material online). However, the estimated admixture dates did not drastically change—17 generations before NAT002 (1,410 BP) for migration of the Amur-related ancestry, and 37 generations before NAT002 (2,010 BP) for admixture between Jomon and Kamchatka lineages—suggesting that our admixture dating had moderate robustness (supplementary fig. S18, Supplementary Material online).

### Genetic Contribution of the Okhotsk People to the Ainu Population

The formation process of the Ainu population has been discussed for a long time. However, although genetic affinities between the Ainu and Jomon individuals have been confirmed in previous studies (Kanzawa-Kiriyama et al. 2017, 2019), no definitive conclusions on the population dynamics after the Neolithic period have been reached. When compared with F23, a PCA plot implied that Ainu individuals were slightly affected by the Northeast Asians (fig. 3), whereas the results of ADMIXTURE analysis indicated that they were mostly represented by a single ancestry (fig. 4). Therefore, we tested *D*(Mbuti, X; F23, Ainu) to verify the genetic influence from the North Asian populations after the Jomon period (fig. 6*b*). As a result, most North Asian populations showed significantly positive *D* values. Especially, the Kamchatka populations, including the Itelmen and Koryak, and the Amur populations, including the Hezhen, Oroqen, Xibo, and Daur, showed strong signals. A previous study reported genetic affinities between the Ainu and Kamchatka populations (Jeong et al. 2016).

**Fig. 8.**
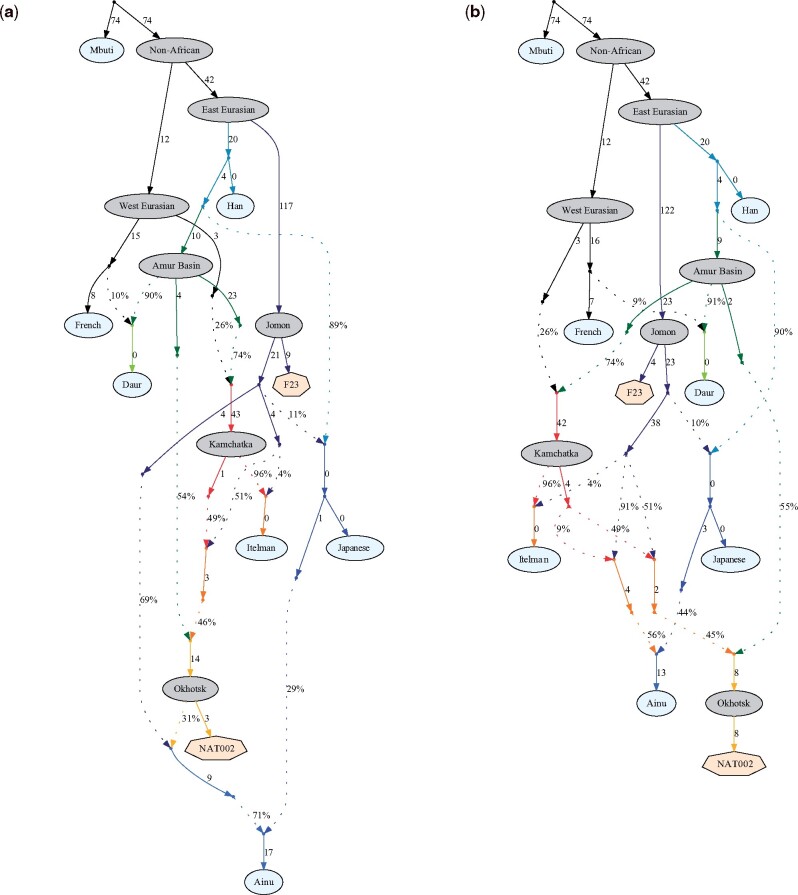
Admixture graphs based on the genomic data of NAT002, F23, and modern populations. Hypothetical ancestral populations are shown by gray ellipses, and ancient genomes are shown by heptagons. Admixture events are shown by dotted arrows. The Z scores for the worst *f*_4_ values in (*a*) the model assuming gene flow from the Okhotsk people to the Ainu and (*b*) the model not assuming gene flow from the Okhotsk people to the Ainu were −2.1 and −2.2, respectively.

To model the formation process of the Ainu population, we constructed admixture graphs using qpGraph (Patterson et al. 2012). First, we constructed an admixture graph involving NAT002, F23, and some SGDP samples (supplementary fig. S19, Supplementary Material online). The order of the admixture events among the Amur, Jomon, and Kamchatka lineages was determined according to the results of the admixture dating. The *Z* score for the worst *f*_4_-statistic in the graph was −2.1. Next, we tested the formation process of the Ainu population by adding the Ainu to the scaffold graph. The model assuming that the Ainu was an admixed population between the Jomon, Okhotsk, and mainland Japanese had the best-fit (*Z* =−2.1, fig. 8*a*) among the models tested. However, another model that did not assume gene flow from the Okhotsk to the Ainu was also acceptable (*Z* =−2.2, fig. 8*b*). Although the first model, which assumed gene flow from the Okhotsk to the Ainu (fig. 8*a*), is the more likely scenario considering the worst *f*_4_ statistic, we could not explicitly reject the second model (fig. 8*b*) in the framework of the admixture graph. One of the notable differences between the first and second models is the admixture ratio from the mainland Japanese to the Ainu (29% in the first model and 44% in the second). Adachi et al. (2018) inferred the admixture ratio of the Jomon, Okhotsk, and mainland Japanese in the early modern Ainu population based on mtDNA haplogroup frequencies. In this study, the genetic contribution of the mainland Japanese to the Ainu was estimated to be 28.1%, which is close to the admixture ratio in our first model, and thus, seems to support the model as the most likely scenario, even though we could not reach a final conclusion.

### Human Leukocyte Antigen Type and Genetic Susceptibility to Hyperostosis of NAT002

Previous studies have reported that the *HLA-B* gene, especially the HLA-B*27 allele, is strongly associated with SAPHO syndrome (Carneiro and Sampaio-Barros 2013). Therefore, to investigate the genetic susceptibility to hyperostosis of NAT002, which was inferred as SAPHO syndrome by a previous study (Okamoto et al. 2016), we determined the human leukocyte antigen (HLA) type of this individual using HLA-VBSeq (Nariai et al. 2015), TARGT (Pierini et al. 2020), and HLA EXPLORE (Omixon, Budapest, Hungary). The *HLA-B* alleles that showed the top two average depths in the typing with HLA-VBSeq were HLA-B*40:02:01 (average depth, 22.93×) and HLA-B*40:06:01:01 (average depth, 12.57×), implying that this individual is a heterozygote of B*40:02:01 and B*40:06:01:01. However, B*40:06:01 allele-specific sequences were not detected by the TARGT and HLA EXPLORE. Probably, B*40:06:01:01 identified with HLA-VBSeq might be a result of a mismapping of the reads derived from *HLA-C* locus because the 100-bp sequence including B*40:06:01-specific substitution sites in *HLA-B* exon 3 shows extremely high identity with the *HLA-C* sequences in IMGT/HLA database release 3.43.0.

The other classical HLA genes were also typed with HLA-VBSeq, TARGT, and HLA EXPLORE. HLA-VBSeq results suggested that NAT002 is a homozygote of A*02:01:01 (average depth, 30.74×), C*15:02:01 (average depth, 33.33×), DQB1*05:03:01 (average depth, 35.07×), and heterozygote of DRB1*14:05:01 (average depth, 7.36×) and DRB1*14:54:01 (12.93×). For *HLA-DRB1*, however, the DRB1*14:05:01 allele was not detected in TARGT and HLA EXPLORE analyses. We also inferred it to be a result of mismapping in HLA-VBSeq analysis. To verify the homozygosity/heterozygosity of NAT002 for HLA region, we investigated alternative allele frequencies in NAT002 sequence data (supplementary fig. S20, Supplementary Material online). Apparent lack of intermediate alternative allele frequencies was observed in the region ranging approximately 10 Mb including HLA region (supplementary fig S20*a*, Supplementary Material online), suggesting a run of homozygosity (ROH). It is unlikely to be allele dropout, considering the similar sequence depths between region which the ROH was observed in and the other regions on chromosome 6. A relatively large number of sites in HLA-DR region including *HLA-DRA*, *-DRB1*, *-DRB5*, and *-DRB6* (for hs37d5, which does not include *HLA-DRB3* and *-DRB4*) showed intermediate alternative allele frequencies, and might be likely to indicate heterozygosity of NAT002 for this region (supplementary fig S20*b*, Supplementary Material online), as also suggested by HLA-VBSeq analysis. However, sequence depths of these sites are remarkably higher than those of the other sites, implying mismapping of sequence reads to this region. Therefore, we finally concluded that NAT002 was homozygote of A*02:01:01, B*40:02:01, C*15:02:01, DQB1*05:03:01, and DRB1*14:54:01, although it would be generally rare that an individual is homozygote for five HLA loci.

Although to our knowledge, the association between SAPHO syndrome and the HLA-B*40 allele has not been sufficiently clarified, Yabe et al. (2010) detected HLA-B61 (serotype including some HLA-B*40 alleles such as B*40:02, B*40:03, and B*40:06) from three of six Japanese patients with SAPHO syndrome. In addition, the HLA-B*40 allele has been reported to be one of the risk alleles for ankylosing spondylitis, reactive arthritis, and undifferentiated spondyloarthropathy, which, similar to SAPHO syndrome, are also diseases with hyperostosis (Arnett et al. 1977; Khan et al. 1978; Khan 1983; Cedoz et al. 1995). Therefore, the HLA type might be able to explain in part the genetic susceptibility to the hyperostosis in NAT002. The HLA-B*40 allele was detected among the Nivkh people at high frequencies: 25.5% in Lou et al. (1998) and 31.2% in the Allele Frequency Net Database (http://www.allelefrequencies.net, last accessed August 25, 2021).

No shared HLA alleles were observed between NAT002 and F23 (homozygote of A*24:02:01, B*15:01:01, and C*03:03:01, which were previously genotyped in Kanzawa-Kiriyama et al. 2019); therefore, from the perspective of the HLA gene, we could not discuss on the genetic relationship between these two individuals, who were excavated from the same island. However, both of these two individuals were regarded as homozygotes for HLA region. This might be attributed to the fact that they probably lived on a very small island, where only small population sizes could be maintained.

### Inferred Phenotypes for Traits Frequently Observed in East/Northeast Asians

We inferred NAT002 phenotypes based on NAT002 genotypes for some SNPs associated with traits showing markedly different frequencies between East/Northeast Asians and the other populations. This individual is a homozygote of the A allele for rs17822931 in *ABCC11*, which determines the earwax phenotype (Yoshiura et al. 2006), suggesting dry earwax. A previous study reported a high frequency of the dry allele (83.9%) among Okhotsk specimens (Sato et al. 2009a). This individual is also a homozygote of the G allele for rs1229984 in *ADH1B* and rs671 in *ALDH2*, which are associated with metabolic rates for alcohol (Edenberg et al. 1997) and acetaldehyde (Harada et al. 1981), respectively, suggesting alcohol tolerance. The alleles for alcohol intolerance show higher frequencies in the populations genetically affected by East Asian Neolithic farmers than in the other populations, including Siberians (Osier et al. 2002; Oota et al. 2004; Koganebuchi et al. 2017). The inferred alcohol tolerance of NAT002 seems to be related to the phenotype frequently observed among Siberian hunter–gatherers rather than East Asian farmers, corresponding to her genetic similarity with Northeast Asian populations.

## Conclusion

We obtained a high-coverage genome of NAT002, a prehistoric Okhotsk individual from the Hamanaka 2 site in northern Japan. The results of our population genetic analyses indicated a close relationship between NAT002 and Lower Amur populations, including the Nivkh and Ulch, which support the results of previous morphological and mitochondrial DNA studies (Ishida 1988, 1996; Komesu et al. 2008; Sato et al. 2009b). The length of the external branch to NAT002 in the phylogenetic tree (fig. 5) was comparable to those to the modern Nivkh and Ulch, implying the small influence of the erroneous genotype call in our sequence data caused by postmortem damage or insufficient depth. The NAT002 genome supports past admixture events among the three lineages (the Amur, Jomon, and Kamchatka) around northern Japan. Among these events, the oldest one seems to have been admixture between the Jomon and Kamchatka lineages. Based on the results of admixture dating, we hypothesized that the admixture between the Jomon and Kamchatka lineages occurred approximately 2,000 BP (corresponding to the Epi-Jomon period). Although to our knowledge, no archaeological evidence suggesting migration from the Kamchatka Peninsula to northern Japan during the Epi-Jomon period has been reported, our results are compatible with those from previous genetic studies (Adachi et al. 2011; Moiseyev et al. 2019). In addition, a previous morphological study based on dental crown measurements also suggested migration from the Asian continent to northern Japan during the Epi-Jomon period, although the source of the migration was assumed to be the Amur Basin instead of Kamchatka (Matsumura 2003). Further genomic studies of Epi-Jomon specimens are required to reveal this admixture event. Migration of the Amur-related ancestry could have occurred approximately 1,600 BP, corresponding to the beginning of the Okhotsk culture as suggested by previous archaeological evidence. Our genomic study support the hypothesis that the migration wave from the Amur Basin was a trigger of the beginning of the Okhotsk culture in this region. On the other hand, we could not reach a final conclusion regarding the genetic contribution of the Okhotsk people to the formation process of the Ainu population.

One of the limitations of this study is to depend on the genome data derived from only one individual (NAT002) of the Okhotsk cultural period, assuming that this individual can be representative the prehistoric Okhotsk people. We cannot completely exclude the possibility that NAT002 is a migrant individual from another region. The carbon and nitrogen isotope ratio indicated that NAT002 fell into the variation of the prehistoric Okhotsk individuals excavated from the same site, suggesting that NAT002 had general food habit of the Okhotsk culture in this region (Okamoto et al. 2016). However, this fact cannot prove that NAT002 was born in this area. Hedges et al. (2007) reported collagen turnover rate of female was 0.060 ± 0.040 during adolescence and 0.041 ± 0.010 after the cessation of growth. Therefore, even if NAT002 was a migrant individual, the food habit before migration would not be reflected in the carbon and nitrogen isotope ratio in the case that sufficient time had passed after the migration. Although strontium isotope analysis might be able to reveal the birthplace of NAT002, the geographic distribution map of strontium isotopes around northern Japan is not available. Another way to investigate whether NAT002 can represent the prehistoric Okhotsk people is to analyze a number of specimens of the Okhotsk individuals. Hence, further genomic studies using multiple Okhotsk specimens are necessary to reveal population history around the Circum-Sea-of-Okhotsk region. Moreover, we assumed single pulse migration model in our admixture dating without any specific basis. In the future, the genomes of multiple individuals from different points of a time transect in the Okhotsk cultural period may reveal their detailed migration style (e.g., single pulse, multiple pulse, or continuous).

In addition to providing suggestions about the human population history around the Circum-Sea-of-Okhotsk region, our research also suggests that DNA molecules longer than 1 kb could survive nearly a thousand years under particular ideal conditions, which might provide additional insights into ancient DNA studies.

## Materials and Methods

### Sampling, DNA Extraction, Library Build, and Sequencing

NAT002 was recovered from the upper surface of the layer corresponding to the final stage of the Okhotsk period. Pieces of the Okhotsk culture-style pottery, iron knives, iron fishing hooks, and metal bracelets, which were believed to be burial items, were excavated from around the skeletal remain of NAT002. Based on the layer of the grave and the style of the pottery excavated from around the skeletal remain, NAT002 was inferred to be an individual at the end of the Okhotsk culture. The result of ^14^C dating was consistent with this inferring (Okamoto et al. 2016).

Whole-genome analysis of NAT002 was approved by the ethics committees of Kanazawa University. We also consulted with the Ainu Association of Hokkaido about the anthropological study of ancient human skeletons recovered from the Hamanaka 2 site.

At the excavation site, the first author (T.S.) dug up and sampled the two-third molars wearing disposable coveralls, a head cap, shoe covers, a face mask, latex groves, and a face shield. Before removing the sand that covered the teeth, all other excavation participants moved away from the grave of NAT002 to reduce the risk of modern DNA contamination. After recovery by digging, the tooth samples were immediately put into separate plastic tubes, and preserved in a deep freezer, and then sent directly to the genetic laboratories at University of the Ryukyus and University of Yamanashi to avoid modern DNA contamination and degradation. Genomic DNA was extracted from two-third molars independently at the different laboratories. One was extracted by T.S. (extract 1) using a silica-based method (Rohland and Hofreiter 2007), and the other was extracted by N.A. (extract 2) using the method of Adachi et al. (2013). The size distributions of the extracted DNA molecules were measured using the Bioanalyzer and High Sensitivity DNA kit (Agilent, Santa Clara, CA). Surprisingly, the extracts, especially extract 2, contained unusually long DNA molecules for an ancient sample (supplementary fig. S1, Supplementary Material online). NGS libraries of extract 2 could not be amplified without artificial DNA shearing because of a lack of short DNA molecules. Therefore, the DNA molecules in extract 2 were sheared using the DNA Shearing System M220 (Covaris, Woburn, MA) to prepare Illumina platform short-read NGS libraries. NGS libraries were prepared without UDG treatment. The end-repair and adapter ligation steps were performed according to the method of Rasmussen et al. (2011), except for the use of 10-fold diluted TruSeq adapters (Illumina, San Diego, CA). PCR amplification was performed for a reaction mixture of 50 µl containing 5 µl of the library, 25 µl of 2× KAPA HiFi HotStart ReadyMix (KAPA Biosystems, Wilmington, MA) for extract 1 or KAPA HiFi HotStart Uracil+ ReadyMix (KAPA Biosystems, Wilmington, MA) for extract 2, 5 µl of primer cocktail in the TruSeq Nano DNA Library Preparation Kit (Illumina, San Diego, CA), and 15 µl of distilled water under the following conditions: 98 °C for 45 s, 12 cycles at 98 °C for 15 s, 60 °C for 3 min, and 72 °C for 30 s, with a final extension at 72 °C for 1 min. The PCR products were purified using the PCR Kleen Spin Column (Bio Rad, Hercules, CA). A total of 18 libraries were prepared, and then shotgun sequencing (100 bp PE) was performed using HiSeq 2000/2500. All procedures during pre-PCR DNA experiments were performed following typical contamination precautions, such as wearing disposable coveralls, head caps, shoe covers, facemasks, latex groves, and face shields. In addition, pre-PCR experiments were carried out in clean benches with positive pressure air filtering, which were installed in dedicated experimental rooms for ancient DNA. These clean benches and rooms were cleaned with DNA-OFF (Takara Bio, Kusatsu, Japan) or DNA-AWAY (ThermoFisher Scientific, Waltham, MA), and the clean benches and laboratory instruments were UV-radiated before and after the DNA experiments. The dedicated ancient DNA room at University of the Ryukyus had been used only by T.S., whereas that at University of Yamanshi had been used by N.A. and one other person before the DNA extraction of NAT002.

### Mapping, Genotype Call, and Filtering

Adapter sequences were trimmed from the obtained sequence reads using AdapterRemoval ver. 2.2.0 (Schubert et al. 2016). Quality control was performed using PRINSEQ-lite (Schmieder and Edwards 2011). We trimmed bases with Phred scores lower than 30 from the 5′ and 3′ ends, and then trimmed one base from the 5′ and 3′ ends to reduce the influence of postmortem deamination. Next, we removed the reads shorter than 35 bp. The sequence reads that passed QC were mapped to the human reference genome (hs37d5) using BWA-MEM (Li and Durbin 2009). The mapping rates were then calculated for each library. Next, the clipped reads and reads with mapping quality lower than 30 were excluded from the BAM files. BAM file processing was performed using SAMTools (Li et al. 2009). Duplicated reads were removed using Picard MarkDuplicates (http://broadinstitute.github.io/picard, last accessed August 25, 2021). After indel realignment and base recalibration using GATK IndelRealigner and BaseRecalibrator, the average depth was calculated using GATK DepthOfCoverage (McKenna et al. 2010). Phred scores were rescaled based on damage level using mapDmage 2.0 (Jonsson et al. 2013).

The following genotype calling and depth-based filtering procedures were performed according to the method of the SGDP (Mallick et al. 2016). First, genotype calling using GATK UnifiedGenotyper with the modified prior was performed to avoid reference bias. Sample-specific CNV filtering was executed using bam2cnv (http://reichdata.hms.harvard.edu/pub/datasets/sgdp/filters/sample_specific/bam2cnv/, last accessed August 25, 2021). The sample specific CNV regions in the reference FASTA were masked using BEDtools ver. 2.25 (Quinlan and Hall 2010). Next, we ran cmakefilter in cTools (Mallick et al. 2016) to conduct the filtering procedure. The divergences to chimpanzee and human references were calculated using filtstats in cTools. We also applied these genotype calling and filtering procedures to the modern Nivkh individuals (Raghavan et al. 2014) and the high-coverage genome of the Jomon individual (F23) excavated from the Funadomari site (fig. 1), which was recently reported by Kanzawa-Kiriyama et al. (2019). The whole-genome sequences of the modern Nivkh individuals were downloaded from the Technical University of Denmark website (http://www.cbs.dtu.dk/suppl/NativeAmerican/. last accessed August 25, 2021). The BAM files were converted to FASTQ files, and then QC and mapping procedures described above were applied to the sequence for the Nivkh individuals before genotype calling and filtering.

### Evaluation of DNA Authenticity

To assess the DNA authenticity of the NAT002 sequence, we confirmed the deamination pattern using mapDamage 2.0 (Jonsson et al. 2013), ANGSD (Korneliussen et al. 2014), and sequence data without trimming the first and last bases of each read. Before merging all the BAM files, we individually called the genotypes from extracts 1 and 2 to confirm their concordance. We called the genotypes for the polymorphic sites observed among the East Asians (JPT and CHB) of the 1000 Genomes Project phase 3 (1000 Genomes Project Consortium 2015) using GATK UnifiedGenotyper under the default settings. The proportion of shared IBD (π^) between the obtained genotype data derived from extracts 1 and 2 was calculated using PLINK (Purcell et al. 2007). The mtDNA-based modern DNA contamination rate was estimated using Schmutzi (Renaud et al. 2015) after remapping the reads to the revised Cambridge Reference Sequence NC_012920.1 (Andrews et al. 1999). The estimated endogenous and contaminant mtDNA haplotypes were assigned to haplogroups using HaploGrep (Weissensteiner et al. 2016). The autosomal DNA-based contamination rates were estimated using DICE (Racimo et al. 2016). CHB was used as an anchor population and JPT as a contaminant population in the 2-Pop method because only Japanese researchers handled the NAT002 specimen at the excavation site and genetic laboratories. To confirm population genetic consistency between read fractions, damaged (PMD score ≥3) and undamaged (PMD score ≤0) sequence reads were extracted from the alignment files from the extracts 1 and 2, using PMDtools (Skoglund et al. 2014). The correlation coefficients between the *f*_3_ values of the NAT002 sequence fractions were calculated using R4.1 (https://www.r-project.org/, last accessed August 25, 2021).

### Population Genetic Analyses

For the population genetic analyses, we prepared three data sets, including different population and SNP sets. For the extraction of polymorphic sites from whole-genome sequence data, we used filtering level 0 when the known SNP sites were extracted, and filtering level 1 when all the variant sites were extracted, according to the recommendation of Mallick et al. (2016). Data set 1 consists of the genotypes of the overlapping SNPs among the previously reported DNA microarray data sets for modern worldwide populations (Rasmussen et al. 2010; Jinam et al. 2012; Fedorova et al. 2013; Lazaridis et al. 2016). From the F23 and NAT002 genomes, we extracted genotypes for the overlapping SNP sites among the above studies using cpulldown in cTools (Mallick et al. 2016) while allowing missing at filtering level 0. Next, we merged genotype data for the modern worldwide populations, F23, and NAT002, and then filtered out the SNP sites with missing rates higher than 2%, resulting in a data set of 49,079 SNPs for 2,244 individuals. Data set 1 was used to carry out an outgroup *f*_3_ test, *D* test, PCA, ADMIXTURE analysis (Alexander et al. 2009), and qpAdm modeling. Because the PCA and ADMIXTURE are not robust against sample size, data from various sources were collected to ensure as many populations (especially Northeast Asian ethnic minorities) and individuals as possible although this procedure reduced the number of SNPs in data set 1. Especially, we considered that detecting the Jomon-related genetic component, which cannot be detected without multisamples of the Jomon and/or Ainu individuals in PCA and ADMIXTURE, was important to overview the genetic feature of NAT002 in this study. Data set 2 consists of the variants observed among the 31 individuals of the SGDP samples, two individuals of the Nivkh (Raghavan et al. 2014), F23, and NAT002. The variant sites at filtering level 1 were extracted using cpoly in cTools, resulting in a data set of 2,556,493 variants for 35 individuals. A phylogenetic tree was constructed based on data set 2 using TreeMix (Pickrell and Pritchard 2012). Constructing a phylogenetic tree is relatively more robust against small sample size than PCA and ADMIXTURE. Therefore, we used whole-genome sequence data set containing 1–3 individuals for each population, to ensure as many variant sites as possible at expense of number of individuals. Data set 3 consists of NAT002, F23, and SGDP samples, along with the array data of modern Japanese and Ainu, genotyped using the Affymetrix 6.0 SNP chip (Jinam et al. 2012). From the genomes of the SGDP samples, F23, and NAT002, we extracted genotypes for the SNP sites using cpulldown in cTools while allowing missing at the filtering level 0. Next, we merged these genotype data and filtered out the SNP sites with missing rates higher than 2%, resulting in a data set of 499,270 SNPs for 329 individuals. Data set 3 was used for local ancestry estimation. Although this analysis requires some SNP density, the data of Ainu individuals were also essential to validate robustness of our local ancestry estimation and admixture dating (supplementary figs. S17 and S18, Supplementary Material online). Therefore, we used overlapping sites of WGS data of SGDP samples, F23, NAT002, and the array data of the Ainu (Jinam et al. 2012), to ensure as many SNP sites as possible while including the Ainu in the data set.

We performed PCA using the Northeast Asian and French populations of data set 1 to detect the individuals recently admixed with European populations (supplementary fig. S21, Supplementary Material online). Some Northeast Asian individuals were plotted between the French and Northeast Asian clusters, so we removed these individuals from data sets 1 and 4 as recent admixed individuals. We also excluded 21 recent admixed Ainu individuals from the data sets 1 and 4 according to the Ainu-15 sample set described in Jinam et al. (2015). In addition, the five further possible admixed Ainu individuals were excluded based on the ADMIXTURE result (fig. 4) from data set 3. *f*_3_ and *D* statistics were calculated using qp3Pop and qpDstat, respectively, in the ADMIXTOOLS package (Patterson et al. 2012). PCA was performed using smartpca in the EIGENSOFT package (Price et al. 2006). The most likely number of *K* in the ADMIXTURE analysis was assessed by 5-fold CV (supplementary fig. S13, Supplementary Material online). The qpAdm modeling was also performed using ADMIXTOOLS package. In this modeling, the Ami, Dai, Mixe, Mbuti, Nganasan, and Papuan were used as outgroups and NAT002 was modeled by three-way admixture (F23-Itelmen-Oroqen). The validity of the proposed model was evaluated based on LRT for the proposed admixture model against full rank model. In addition, we explored whether a two-way admixture model can adequately explain the NAT002 genome based on *P* values for nested models. *P* values for the nested models were calculated using R 4.1 based on the difference in chi-squares and the difference in degrees of freedom between each two-way admixture model and three-way admixture model. Haplotype phasing for data set 3 was performed using SHAPEIT2 (Delaneau et al. 2013) based on the phased data and genetic map of the 1000 Genomes Project Phase 3 (1000 Genomes Project Consortium 2015). Local ancestries were inferred using RFMix (Maples et al. 2013) and ancestry_pipeline (Martin et al. 2017). The SGDP Even, Daur, Hezhen, Oroqen, Xibo, and Mongola were used as the Amur reference, F23 (and Ainu in the replication analysis) as the Jomon reference, and the SGDP Itelman, Chukchi, Eskimo-Chaplin, Eskimo-Naukan, and Eskimo-Sireniki as the Chukot-Kamchatka reference. Haplotype blocks assigned to ancestries with posterior probabilities higher than 0.9 were used for subsequent tract length analysis. Admixture dating was performed using Tracts (Gravel 2012) assuming the three-population single pulse model. The admixture graphs were constructed using qpGraph in ADMIXTOOLS (Patterson et al. 2012). At first, we started from the four-population tree including the Mbuti, French, F23, and Han. Next, we added Itelman as an admixed population between the Northeast Asian and West Eurasian populations. Then, NAT002, Daur, and Japanese were added to the graph. Finally, Ainu was added to the graph.

### HLA Typing

We determined the HLA type of this individual using HLA-VBSeq (Nariai et al. 2015), TARGT (Pierini et al. 2020), and HLA EXPLORE (Omixon, Budapest, Hungary). Because inconsistent results were obtained for *HLA-B* (heterozygote of B*40:02 and B*40:06 or homozygote B*40:06) and *HLA-DRB1* (heterozygote of DRB1*14:05:01 and DRB1*14:54:01 or homozygote of DRB1*14:54:01) between HLA-VBSeq and the other two programs, we checked the alignment results of these loci with BLAST search using IMGT/HLA database release 3.43.0. To confirm the homozygosity/heterozygosity of NAT002 for HLA region, we investigated alternative allele frequencies at 1KG variant sites sequenced with depths ≥15× in NAT002 sequence data.

## Supplementary Material

Supplementary data are available at *Genome Biology and Evolution* online.

## Supplementary Material

evab192_Supplementary_Data
