# Supplementary material for: Whole-Genome Sequencing of a 900-Year-Old Human Skeleton Supports Two Past Migration Events from the Russian Far East to Northern Japan
Source: Genome Biol Evol. 2021 Aug 19;13(9):evab192. doi: 10.1093/gbe/evab192 (PMC8449830; doi:10.1093/gbe/evab192)
Supplement: evab192_Supplementary_Data [file evab192_supplementary_data.pdf]

Supplementary Text S1 Authenticity of the obtained NAT002 genome sequence

The DNA length distributions of the two obtained extracts from NAT002, especially extract 2, were unusually long as ancient DNA molecules (supplementary fig. S1). Although these DNA length distributions made us suspect modern DNA contamination, the same mtDNA (assigned to haplogroup G1b) sequence and STR profile were obtained from the two extracts by Sanger sequencing for the D-loop region and autosomal STR typing. The obtained D-loop sequence was different from those of the dedicated ancient DNA room users at University of the Ryukyus (T.S.) and in University of Yamanashi (N.A. and one other person). At the archaeological site where NAT002 was excavated, only the first author (T.S.) handled the tooth samples for ancient DNA analysis (see the Materials and Methods). Given these facts, it seems premature to interpret the long DNA fragments obtained from NAT002 specimens as simply derived from modern DNA contamination.

We performed next-generation sequencing (NGS) to confirm the existence of the postmortem damage pattern and the genome-wide sequence concordance between the two extracts. To prepare the Illumina platform NGS libraries, DNA molecules in extract 2 were artificially sheared because of their DNA length (see the Materials and Methods). To confirm the sequence concordance between extracts 1 and 2, the proportion of identity-by-descent (IBD), i.e., $\hat{\pi}$, between the genotypes from the two extracts, which were called using the GATK UnifiedGenotyper under the default settings, was calculated based on 7,698,470 polymorphic sites (MAF $\geq$ 0.01) observed among JPT and CHB in the 1000 Genomes Project (The 1000 Genomes Project Consortium 2015). Theoretically, monozygotic twins and parent–child/sibling pairs indicate $\hat{\pi}$ values of 1 and 0.5, respectively. The results showed a passably high proportion of IBD ($\hat{\pi} = 0.9308$),

although the two extracts were obtained by distinct experimenters and showed remarkably different DNA length distributions (supplementary fig. S1), which suggested that majority of the sequence data from extracts 1 and 2 were derived from the same person. Discordant genotypes between the extracts might be explained by insufficient depth, especially extract 1 (extract 1: 9.88×, extract 2: 25.15×).

In addition, sequence data derived from both of the extracts showed a postmortem deamination pattern (supplementary fig. S2). The deamination rates of extract 2 must be underestimated because of artificial DNA shearing for library preparation. Asymmetric damage rates between the 5' and 3' ends of both extracts seem to be explicable by the relationship between insert length and read length (maximum read length of 100 bp). In fact, read fractions from extract 1 containing inserts of 100 bp or shorter showed almost symmetric deamination rates, and only the fraction containing inserts longer than 100 bp showed asymmetric deamination rates (supplementary fig. S3). In addition, similar deamination rates at the 5' end for all fractions from extract 1 suggest that the ratio of endogenous/exogenous DNA in extract 1 is independent of the insert length. Because extract 2 was artificially sheared, we did not divide the sequence data based on the insert size.

The estimated contamination rates based on mtDNA data were only 1% for both extracts (supplementary fig. S4). The estimated endogenous mtDNA haplotypes of both extracts were assigned to haplogroup G1b, which is consistent with preliminary Sanger sequence results and commonly observed among the prehistoric Okhotsk specimens previously analyzed (Sato et al. 2009). The estimated contaminant haplotypes possessed hundreds of private mutations, although these were assigned to haplogroup H2a2a, to which the revised Cambridge Reference Sequence (Andrews et al. 1999) belongs.

MtDNA haplotypes of users for the dedicated ancient DNA rooms at University of the Ryukyus (T.S.) and University of Yamanashi (N.A. and one other person) did not belong to these haplogroups. Therefore, the estimated contaminant seemed to be artificial haplotypes constructed from sequence reads with sequence errors or postmortem damage.

Similarly, the estimated contamination rates based on autosomal DNA data were also sufficiently low for both extracts. The contamination rate based on chromosome 1, for instance, was estimated at 0.5% for extract 1 (supplementary fig. S5) and 0.06% for extract 2 (supplementary fig. S6), assuming that the contaminant DNA was derived from modern Japanese (1KG JPT). Substantially low contamination rates were obtained from the other chromosomes, and the highest contamination rates (0.78%) were observed for chromosome 3 for extract 1 (supplementary fig. S5).

As further analyses to confirm the authenticity of the DNA, we performed preliminary population genetic analyses using read fractions based on insert length or the presence/absence of postmortem damage. To do this, adding to the four fractions from extract 1 based on insert length (supplementary fig. S3), we obtained four fractions of sequence data based on PMD scores (Skoglund et al. 2014). Sequence data derived from both extracts were divided into damaged reads (PMD score ≥ 3) and undamaged reads (PMD score ≤ 0). For all fractions of the sequence data, we called pseudo-haplotypes by random sampling of a single read at each SNP site in dataset 1 (see the Materials and Methods) using pileupCaller in SequenceTools (https://github.com/stschiff/sequenceTools).

PCA was performed using F23, modern East, and Northeast Asian populations in dataset 1, including 49,079 SNP sites, and then all NAT002 read fractions were projected on the surface of eigenvectors 1 and 2. As a result of the PCA (supplementary fig. S7a),

basically all NAT002 fractions were plotted close to each other, suggesting that the majority of insert DNA in all fractions was derived from the same population. Only a fraction of 35–50 bp inserts was plotted at a distance from the other fractions, which could probably be attributed to the low coverage of the fraction (0.06×), not the differences in the genetic background. To confirm this, we generated 100 datasets that were down-sampled from the other read fractions to achieve uniform coverage between all fractions, and then performed PCA again (supplementary fig. S7b). The results indicated that the down-sampled read fractions were slightly shifted from their original positions on the eigenvector surface and the fraction of 35–50 bp fell into the variation of the other down-sampled read fractions, implying that there is no significant genetic differentiation between 35–50 bp and the other fractions.

We also calculated f_3(Mbuti;NAT002,X) for all NAT002 read fractions. The modern East and Northeast Asian populations (Ainu, Buryat, Chukchi, Daur, Eskimo, Even, Evenk, Han, Hezhen, Itelmen, Japanese, Koryak, Mongola, Nivkh, Oroqen, Ulch, Xibo, Yakut, and Yukagir) were used as population X. All pairs of fractions showed highly correlated f_3 values (supplementary fig. S8). Incidentally, the correlation coefficients between the f_3 values of Japanese, which would be the most likely contaminant source population in this study (because NAT002 tooth samples were handled by only Japanese males and all ancient DNA room users were also only Japanese males), and all NAT002 read fractions ranged from 0.31 to 0.58, which were drastically lower than those between the NAT002 read fractions, and were not statistically significant after the Bonferroni correction.

In general, it is believed that DNA molecules longer than 100 bp rarely survive for 100 years (Sawyer et al. 2012). Therefore, long DNA molecules such as those

observed in NAT002 DNA extracts have usually been regarded as modern DNA contamination. However, considering the observed high $\hat{\pi}$ value between extracts 1 and 2, the estimated low contamination rates (supplementary figs. S4, S5, and S6), and the consistent results of preliminary population genetic analyses using read fractions (supplementary figs. S7 and S8), the majority of sequence reads from both of the extracts are likely to be derived from a single individual. Also, the similar deamination rates across the read fractions from extract 1 based on insert length contradict the possibility that the obtained extracts were a mixture of short endogenous and long contaminant DNA molecules.

One possible explanation for the long DNA molecules showing the deamination pattern and high degree of sequence concordance observed in the NAT002 sequence data is "old" modern DNA contamination, which can sometimes occur in ancient samples that have been preserved for several decades in museums or institutes. However, this also seems unlikely, considering the situation when the NAT002 teeth were sampled. After the excavation, the tooth samples were immediately preserved in a freezer and then directly sent to the genetic laboratories, without even washing (see the Materials and Methods); therefore, these samples correspond to "virgin" samples described in Pilli et al. (2013). In such a situation, there is no opportunity for "old" modern DNA showing a deamination pattern to contaminate the samples.

In addition, Sawyer et al. (2012) reported that 100-year-old or younger samples tended to show greater A than G overrepresentation at position –1. For NAT002 extract 1, the frequency of G at position –1 was approximately 0.1 higher than those at positions –10 to –5, and the frequency of A at position –1 was approximately 0.02 higher than those at positions –10 to –5 (supplementary fig. S2). This pattern is similar to those of samples

older than 500 years in Sawyer et al. (2012), the ages of which were consistent with the ^{14}C age of NAT002 (*c*. 900 BP). Moreover, overrepresentation of G at position –1 was more remarkable than that of A, even for extract 2, although it was artificially sheared, probably partially reflecting the original fragmentation pattern before the artificial shearing.

Based on the facts described above, we concluded that the majority of the obtained sequence data were derived from authentic NAT002 DNA. To our knowledge, this paper is the first report implying the possibility that thousands of bp of DNA molecules could survive for hundreds of years under particular ideal conditions. Although it is unclear why the NAT002 DNA was miraculously well preserved, we speculate some possible reasons as follows.

First, the Hamanaka 2 site, from which NAT002 was excavated, is a shell midden on a sand dune on the coast. A sand dune has good drainage, so the water that causes DNA degradation does not stay in the site for a long time. In addition, the bacterial activity in sand seems to be lower than that in soil because of a lack of organic matter. The large amount of sea shells, which contain calcium carbonate, buried in the site could have neutralized the acid (Waselkov 1987), thereby protecting archeological relics derived from organisms, including bones, teeth, and the nucleic acid within them; thus, such a burial environment might have delayed the DNA degradation of NAT002.

Second, the site is located on an island in the northernmost part of the Japanese Archipelago, and the climate of this island belongs to subarctic zone. In fact, alpine plants grow naturally in the lowlands of this island. Moreover, according to Soil Inventory of Japan (https://soil-inventory.dc.affrc.go.jp/) (Takata et al. 2011), the average soil temperature around the excavation site is 8°C at 30–50 cm below the surface, which

corresponds to a border between “Frigid” and “Mesic” defined by United States Department of Agriculture soil taxonomy. Such a low temperature also seemed to help DNA survival in the NAT002 skeleton. Of course, the temperature of this site must be higher than those in permafrost, from which some ancient genomes have been reported (e.g., Raghavan et al. 2014; Harney et al. 2019), and those DNA lengths were much shorter than those of NAT002. However, the samples used in Raghavan et al. (2014) and Harney et al. (2019) had been preserved in a museum or institute; thus, it is not strange that those samples had more fragmented DNA.

Third, NAT002 is not very old as a subject of an ancient DNA study. Although the previous study (Sawyer et al. 2012) evaluated damage patterns of ancient DNA samples of various ages, it appears that there is still insufficient knowledge about DNA survival when 900-year-old ancient human bones are carefully treated for DNA analysis from the excavation stage. In fact, Sawyer et al. (2012) used museum samples, including bones preserved in museums for several decades, to evaluate temporal damage patterns in ancient DNA. Of course, this study provided valuable indicators, while they might have underestimated the degree of DNA fragmentation during preservation in museums and overestimated that in burial environments.

References

Andrews RM, Kubacka I, Chinnery PF, Lightowlers RN, Turnbull DM, Howell N. 1999. Reanalysis and revision of the Cambridge reference sequence for human mitochondrial DNA. *Nat Genet.* 23:147.

Harney É, Nayak A, Patterson N, Joglekar P, Mushrif-Tripathy V, Mallick S, Rohland N, Sedig J, Adamski N, Bernardos R, et al. 2019. Ancient DNA from the skeletons of Roopkund Lake reveals Mediterranean migrants in India. *Nat Commun.* 10:3670.

Pilli E, Modi A, Serpico C, Achilli A, Lancioni H, Lippi B, Bertoldi F, Gelichi S, Lari M, Caramelli D. 2013. Monitoring DNA contamination in handled vs. directly excavated ancient human skeletal remains. *PLos One* 8:e52524.

Raghavan M, Skoglund P, Graf KE, Metspalu M, Albrechtsen A, Moltke I, Rasmussen S, Stafford TW, Jr., Orlando L, Metspalu E, et al. 2014. Upper Palaeolithic Siberian genome reveals dual ancestry of Native Americans. *Nature* 505:87–91.

Sato T, Amano T, Ono H, Ishida H, Kodera H, Matsumura H, Yoneda M, Masuda R. 2009. Mitochondrial DNA haplogrouping of the Okhotsk people based on analysis of ancient DNA: an intermediate of gene flow from the continental Sakhalin people to the Ainu. *Anthropol Sci.* 117:171–180.

Sawyer S, Krause J, Guschanski K, Savolainen V, Pääbo S. 2012. Temporal patterns of nucleotide misincorporation and DNA fragmentation in ancient DNA. *PLoS One* 7:e34131.

Takata Y, Kuwagata T, Kohyama K, Obara H. 2011. Delineation of Japanese soil temperature regime map. *Soil Sci Plant Nutr.* 57:294–302.

The 1000 Genomes Project Consortium 2015. A global reference for human genetic variation. *Nature* 526:68–74.

Waselkov GA. 1987. Shellfish gathering and shell midden archaeology. In: Schiffer M, editor. Advances in archaeological method and theory, volume 10. Cambridge: Academic Press. p. 93–171.

Supplementary Table S1 Number of mapped reads to each chromosome.

Chromosome	Size	Number of mapped reads
mtDNA	16571	99871
chr1	249250621	25180192
chr2	243199373	23942474
chr3	198022430	18907234
chr4	191154276	16400175
chr5	180915260	16823849
chr6	171115067	15971540
chr7	159138663	16128170
chr8	146364022	14217791
chr9	141213431	11922626
chr10	135534747	15411588
chr11	135006516	14165827
chr12	133851895	13648586
chr13	115169878	8555923
chr14	107349540	9154600
chr15	102531392	9032457
chr16	90354753	10269982
chr17	81195210	10274198
chr18	78077248	7566543
chr19	59128983	8427712
chr20	63025520	7666622
chr21	48129895	3755527
chr22	51304566	4975535
chrX	155270560	13578314
chrY	59373566	215317

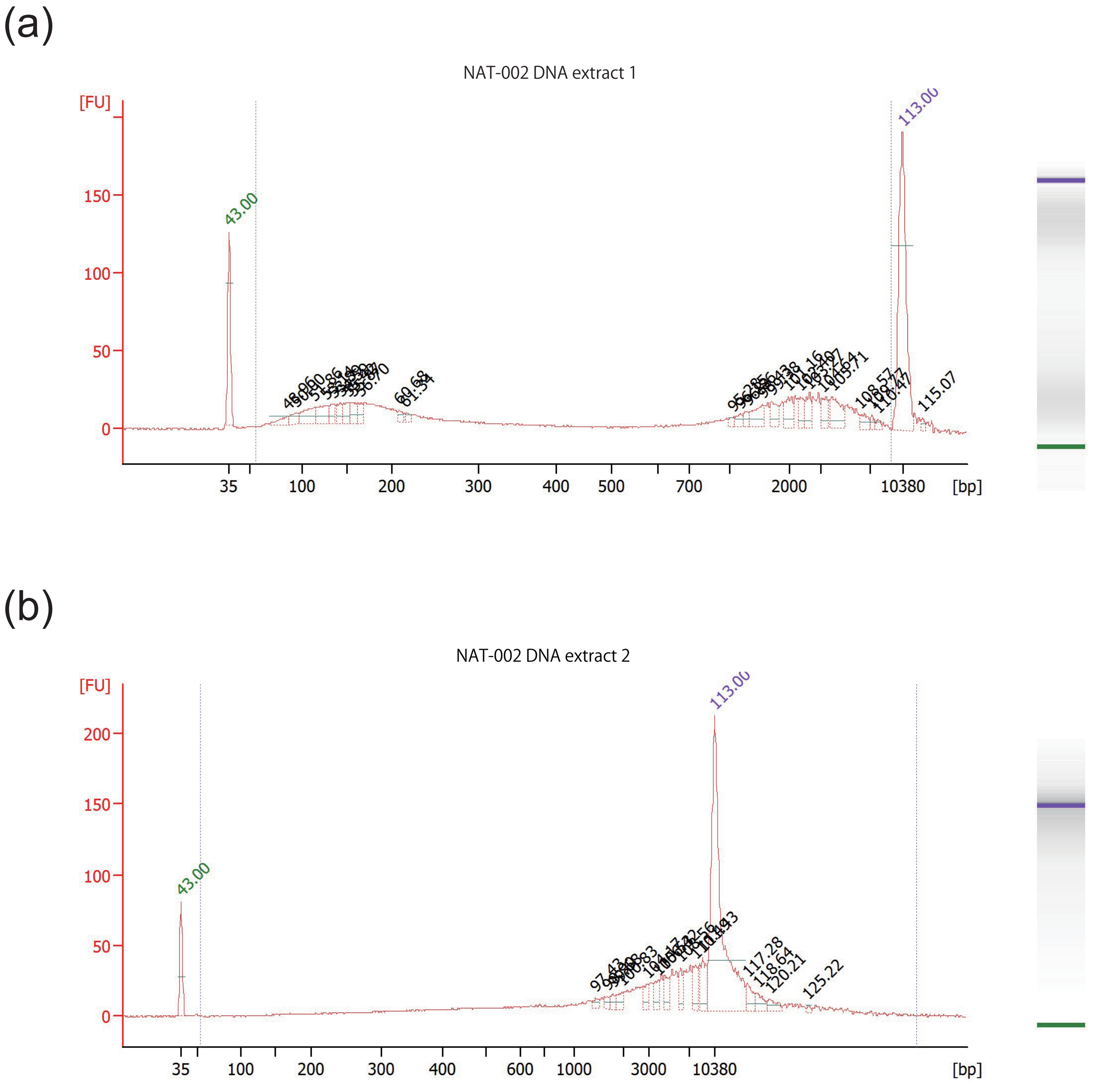

Supplementary Figure S1 Results of electrophoresis for DNA extracts from NAT002 tooth samples. **(a)** Extract 1 was obtained by T.S. according to the method of Rohland and Hofreiter (2007). **(b)** Extract 2 was obtained by N.A. according to the method of Adachi et al. (2013).

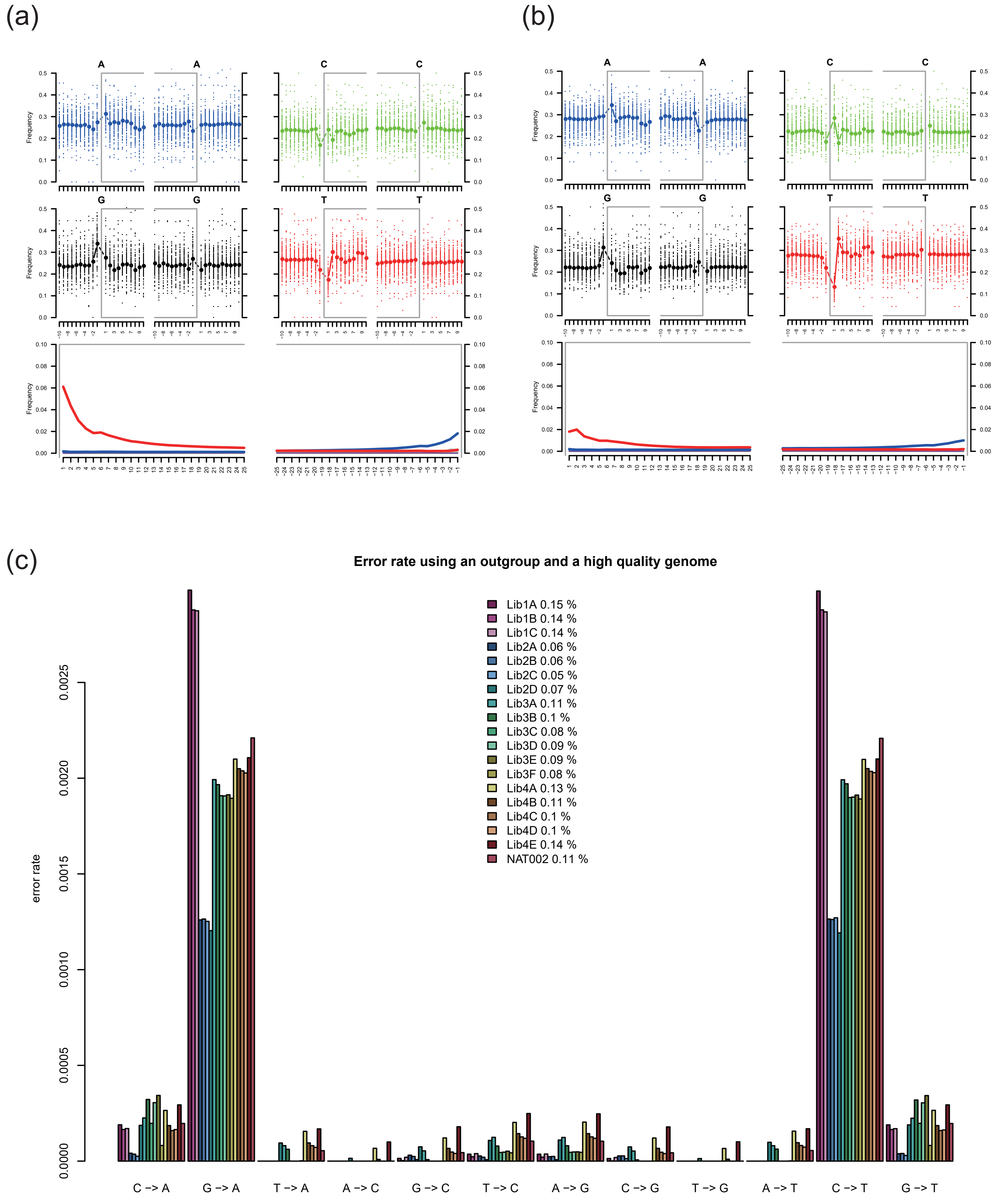

Supplementary Figure S2 Depurination and deamination patterns of the NAT002 sequence. **(a)** Damage patterns observed in extract 1. **(b)** Damage patterns observed in extract 2. The deamination level at the 3’ end was approximately one-third that at the 5’ end, probably because of the abundant reads derived from inserts more than 100 bp. **(c)** Error rates of each library and merged data estimated using PanTro2 and a high-quality modern Japanese genome. NAT002 indicates the merged data of NAT002 alignment files. Lib 1A, Lib1B, and Lib1C were derived from extract 1. The other libraries were derived from extract 2.

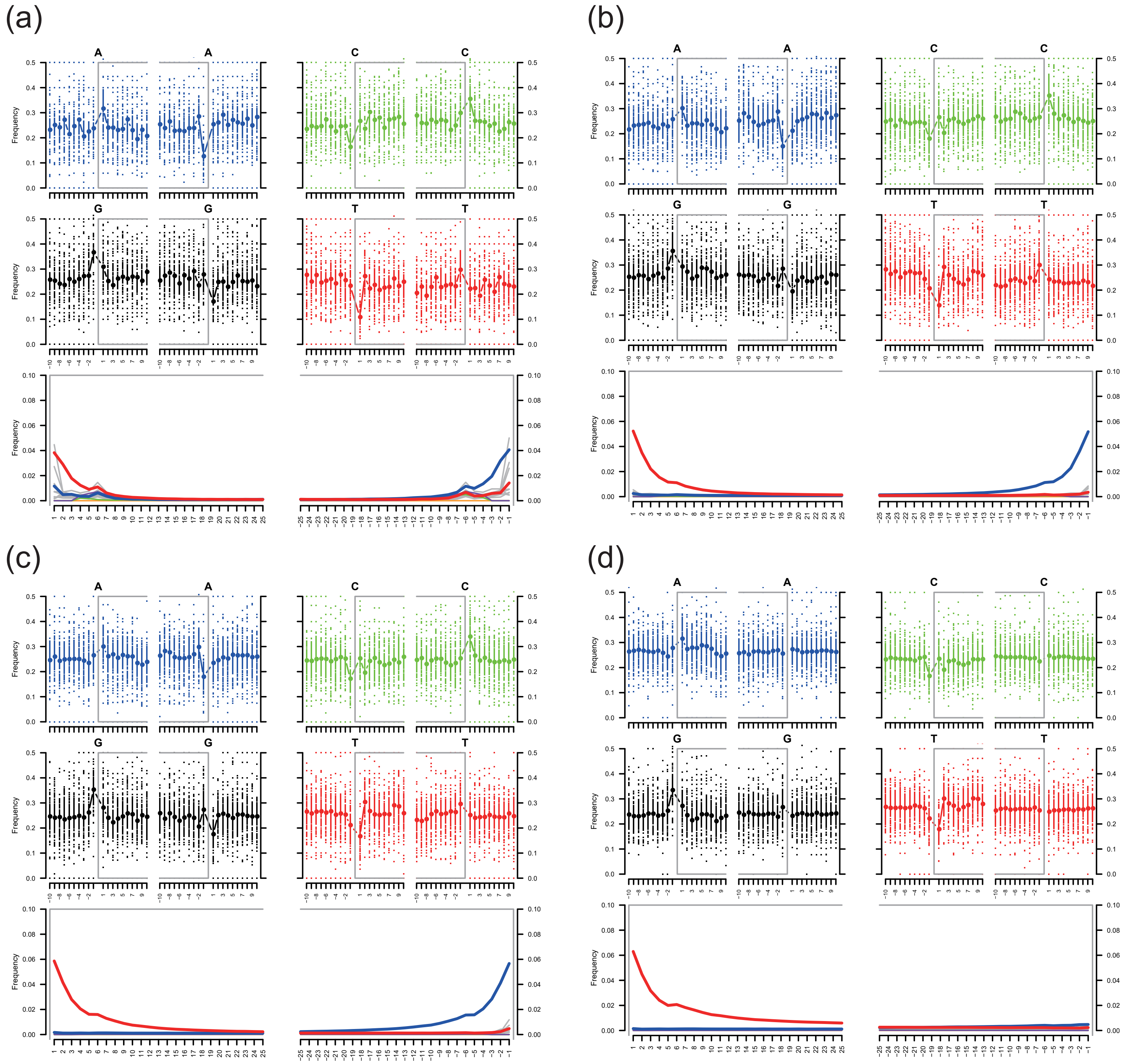

Supplementary Figure S3 Depurination and deamination patterns observed in each fraction based on the insert length of extract 1. (a) 35-50 bp, (b) 51-75 bp, (c) 76-100 bp, and (d) over 100 bp. Asymmeric deamination rates between the 5’ and 3’ ends in (d) can be explained by the relationship between insert and read lengths.

(a)

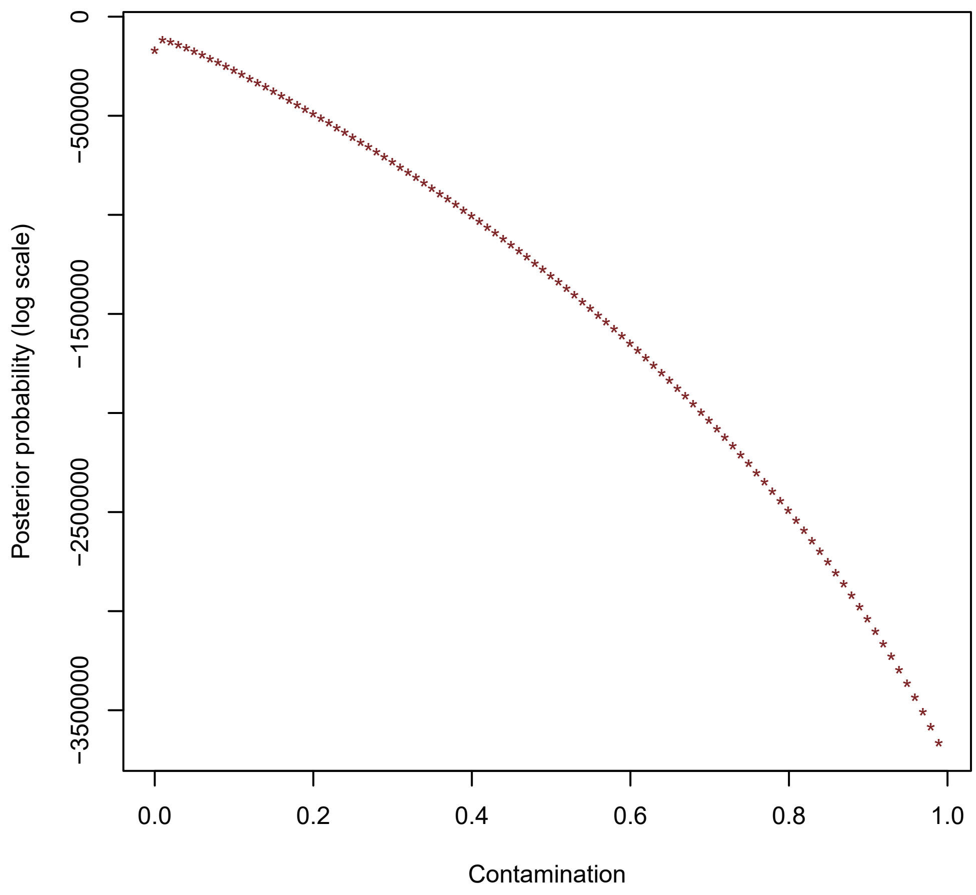

(b)

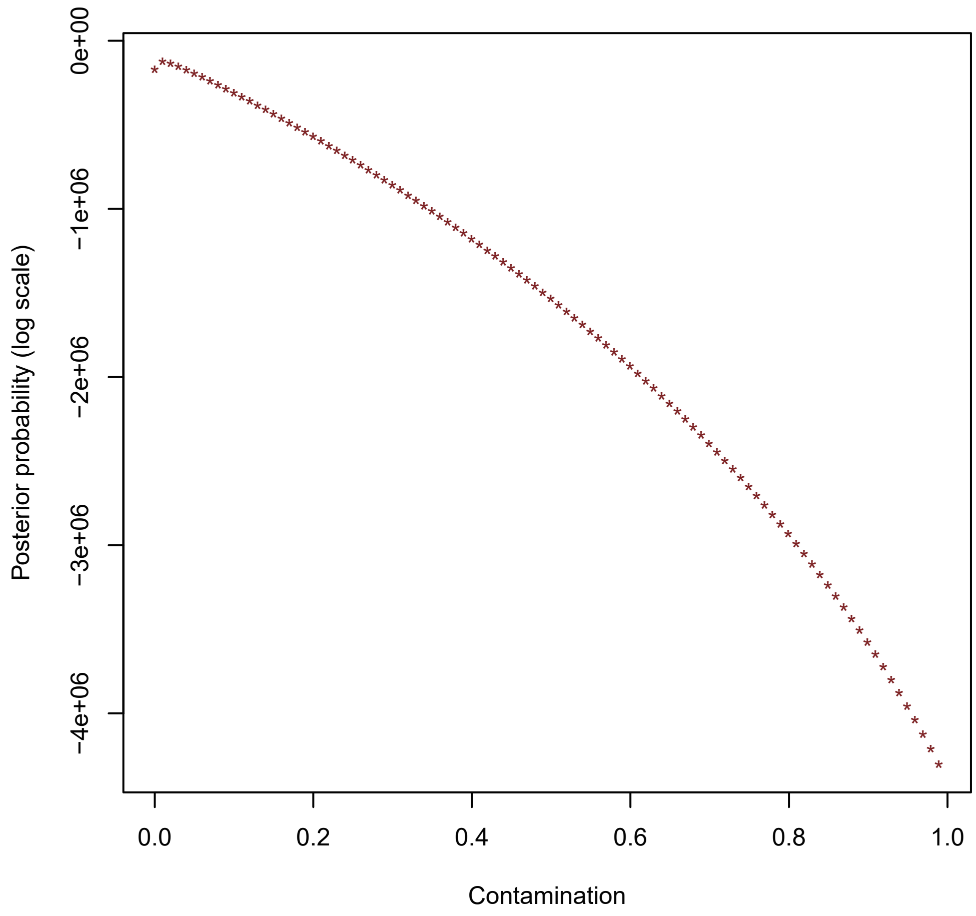

Supplementary Figure S4 Posterior probability for the modern DNA contamination rate inferred from mtDNA sequence data derived from **(a)** extract 1 and **(b)** extract 2.

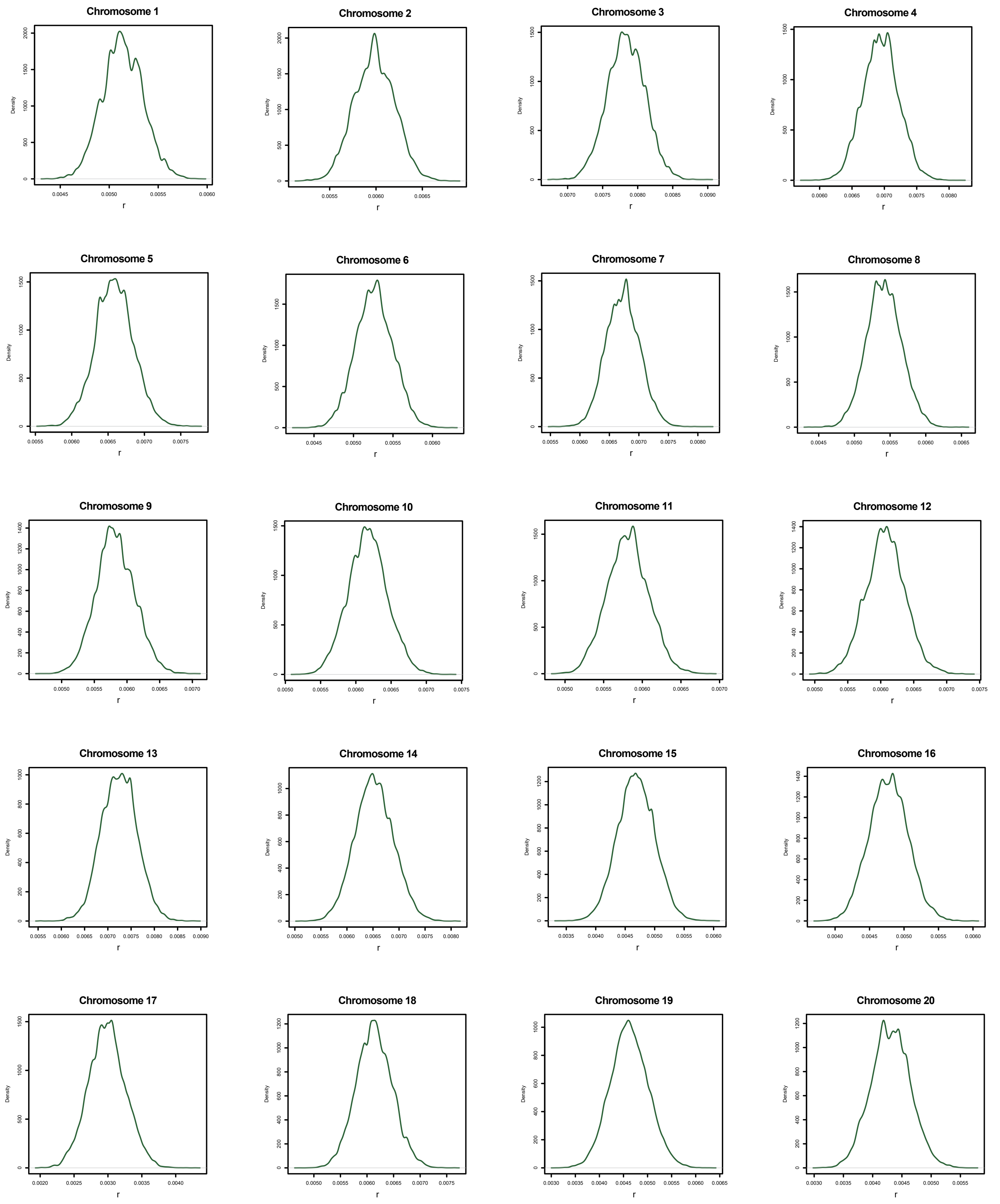

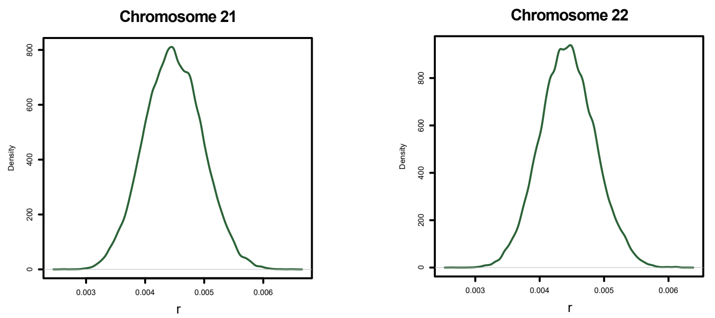

Supplementary Figure S5 Posterior probabilities for the contamination rate (r) of extract 1 estimated using the 2-pop method. 1KG CHB and JPT were used as anchor and contaminant populations, respectively.

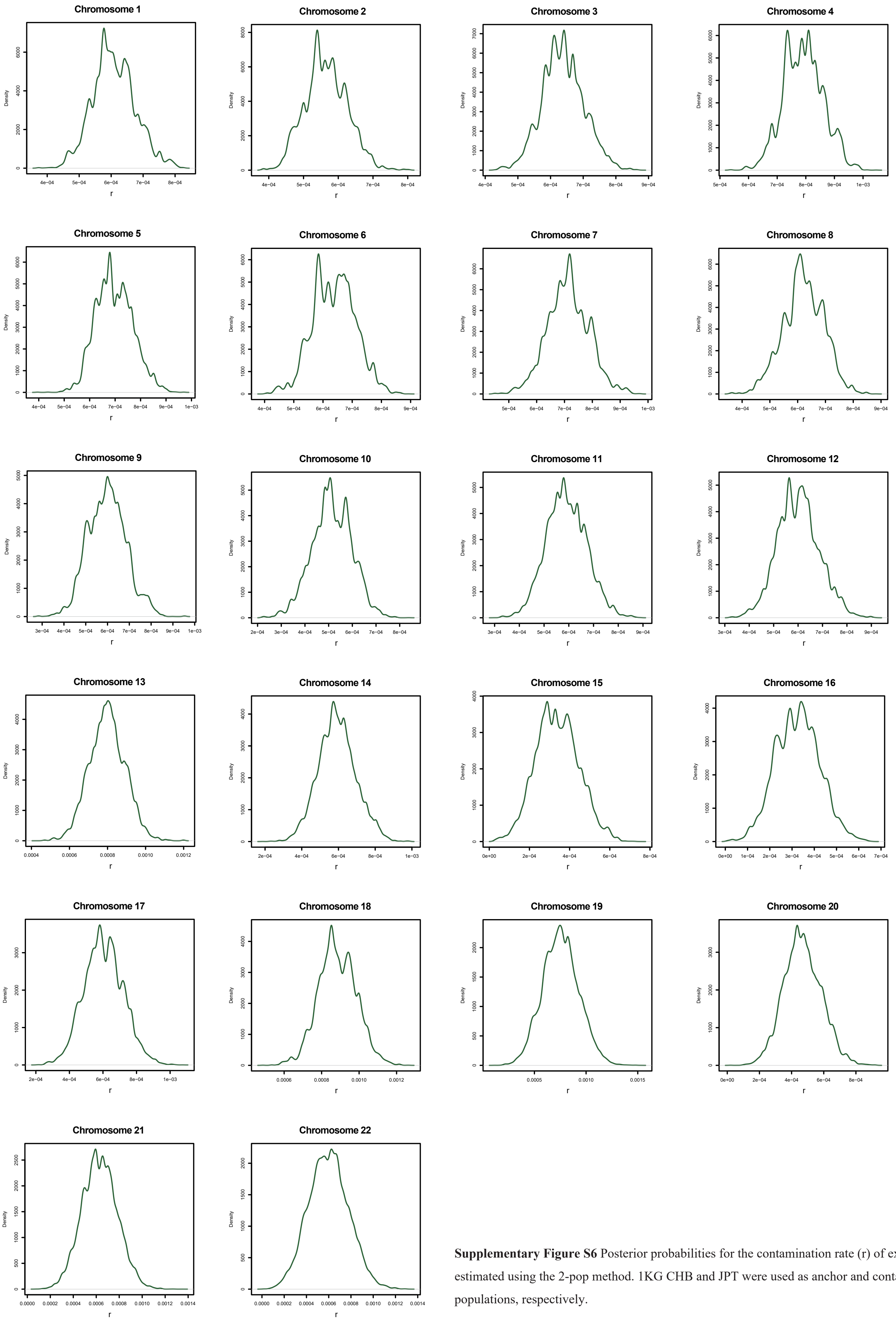

Supplementary Figure S6 Posterior probabilities for the contamination rate (r) of extract 2 estimated using the 2-pop method. 1KG CHB and JPT were used as anchor and contaminant populations, respectively.

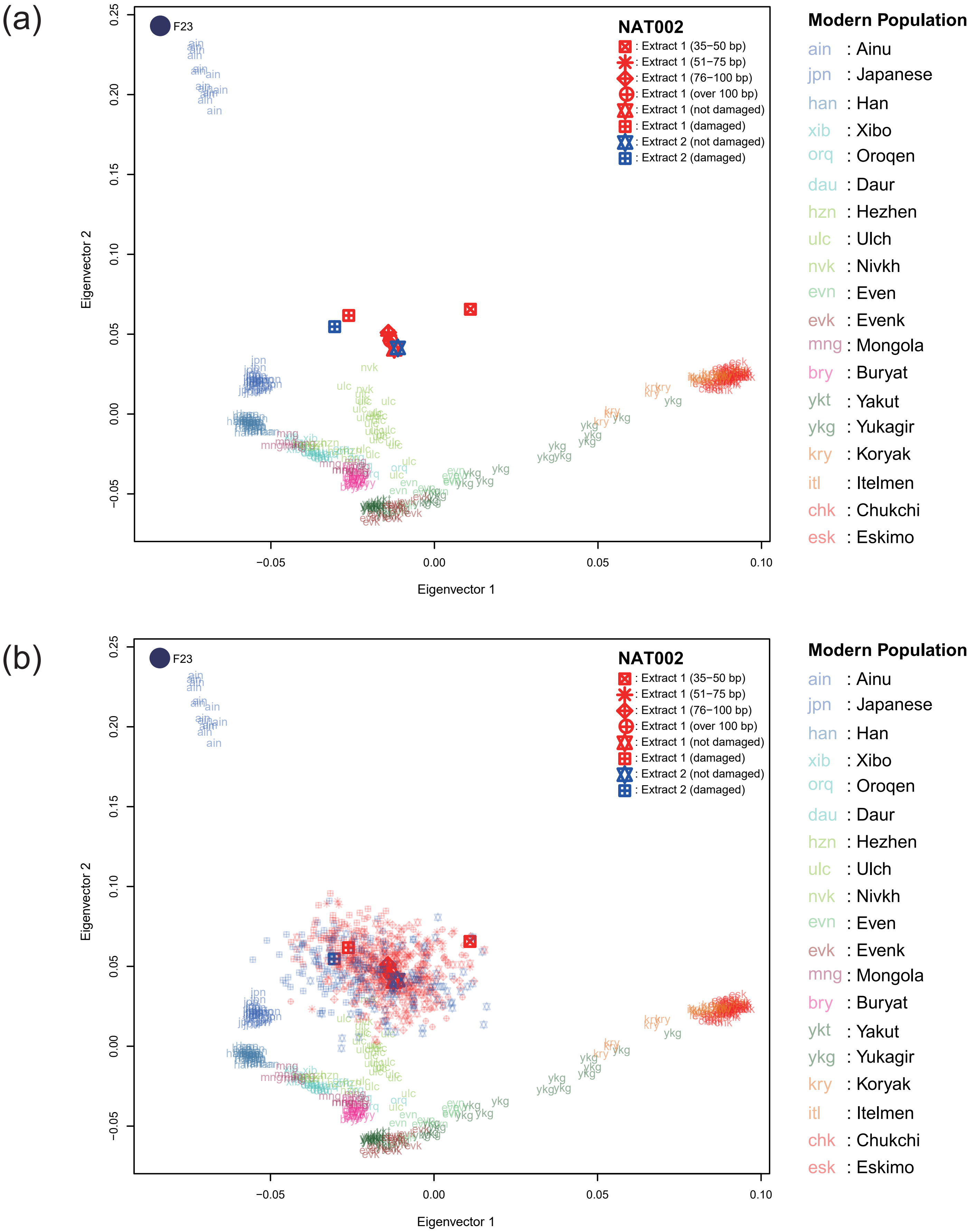

Supplementary Figure S7 PCA for fractions of the NAT002 sequence reads based on insert length or PMD score. In this analyis, “damaged” reads were defined as reads with a PMD score ≥ 3, and “undamaged” reads were defined as reads with a PMD score ≤ 0. All fractions were projected on a surface based on eigenvectors 1 and 2. **(a)** All reads for each fraction were used. **(b)** In additon to full data for each read fraction, reads which were down-sampled to make uniform coverage between fractions (0.06×) were also plotted. Small semipermeable symbols for NAT002 indicate down-sampled read fractions.

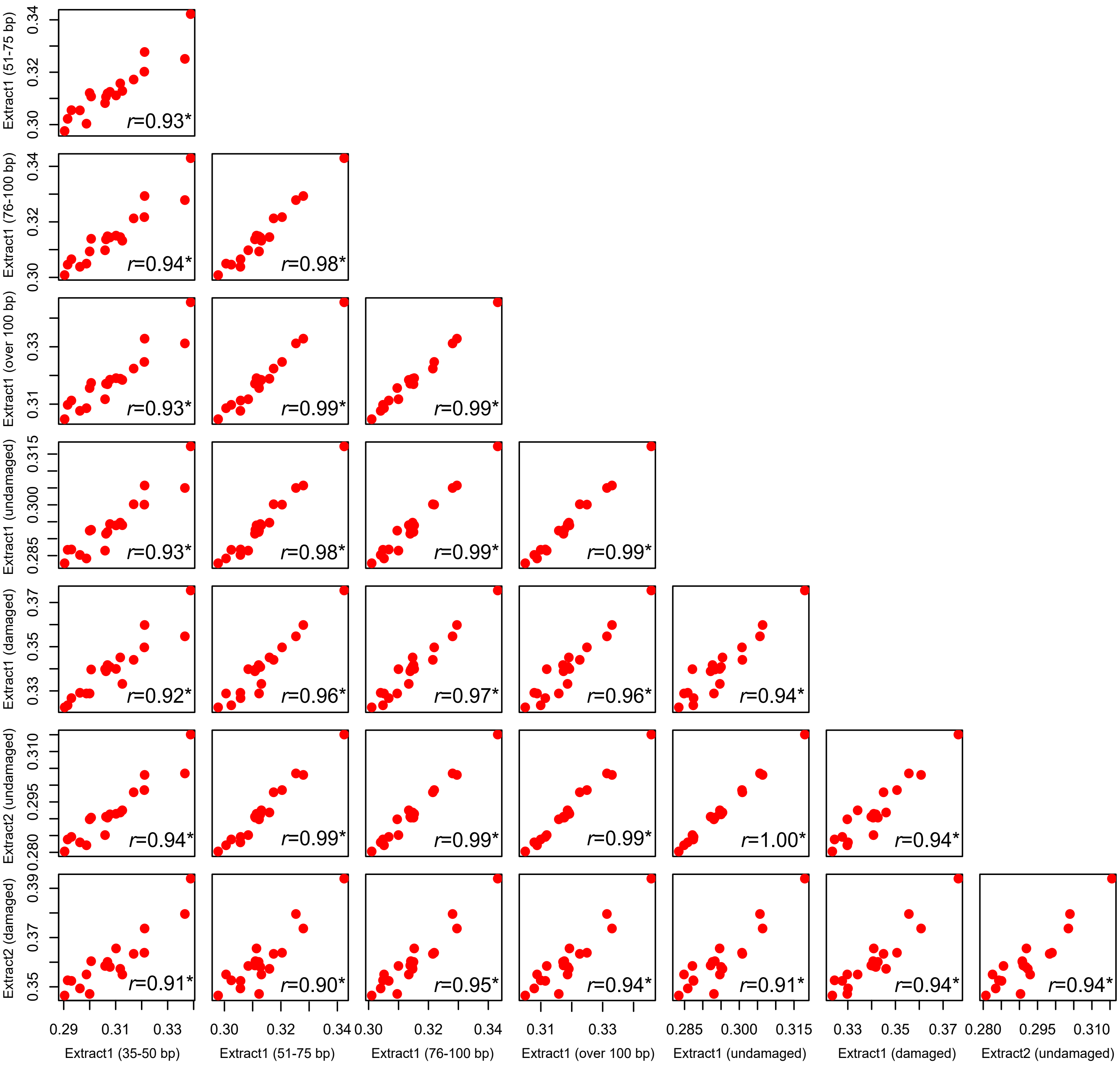

Supplementary Figure S8 Correlation between outgroup f_3 values of all read fractions of the NAT002 sequence. f_3(Mbuti;NAT002,X) was calculated for each fraction. The Ainu, Japanese, Han, Buryat, Chukchi, Daur, Eskimo, Even, Evenk, Hezhen, Itelmen, Koryak, Mongola, Nivkh, Oroqen, Ulch, Xibo, Yakut, and Yukagir were used as population X. Pearson's correlation coefficients were calculated for all pairs of read fractions. The correlation coefficients shown in the figure were rounded off to the second decimal place. Asterisks indicate a statistically significant correlation (P<0.05) after the Bonferroni correction.

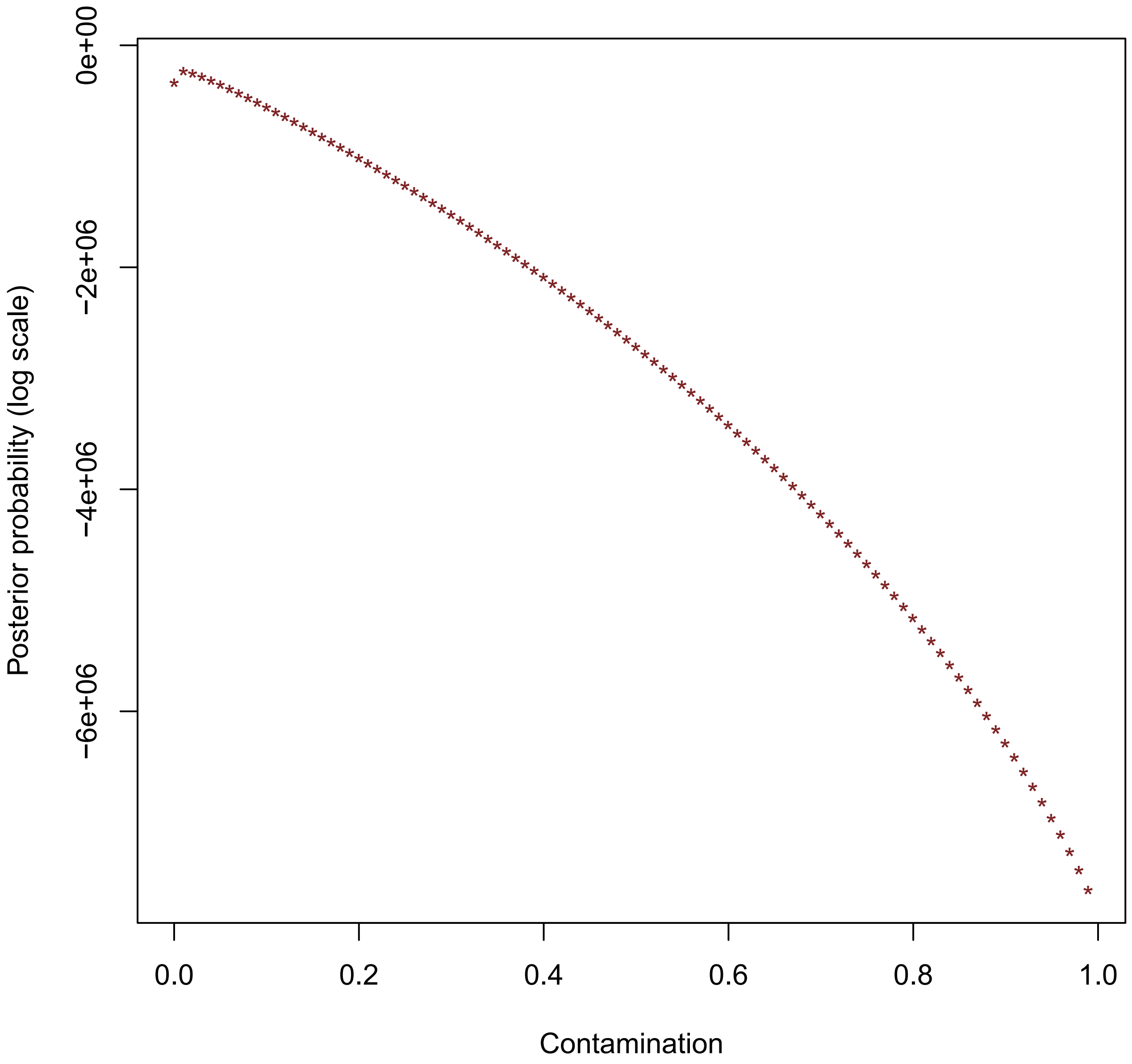

Supplementary Figure S9 Posterior probability for the modern DNA contamination rate inferred from mtDNA sequence data after merging extracts 1 and 2.

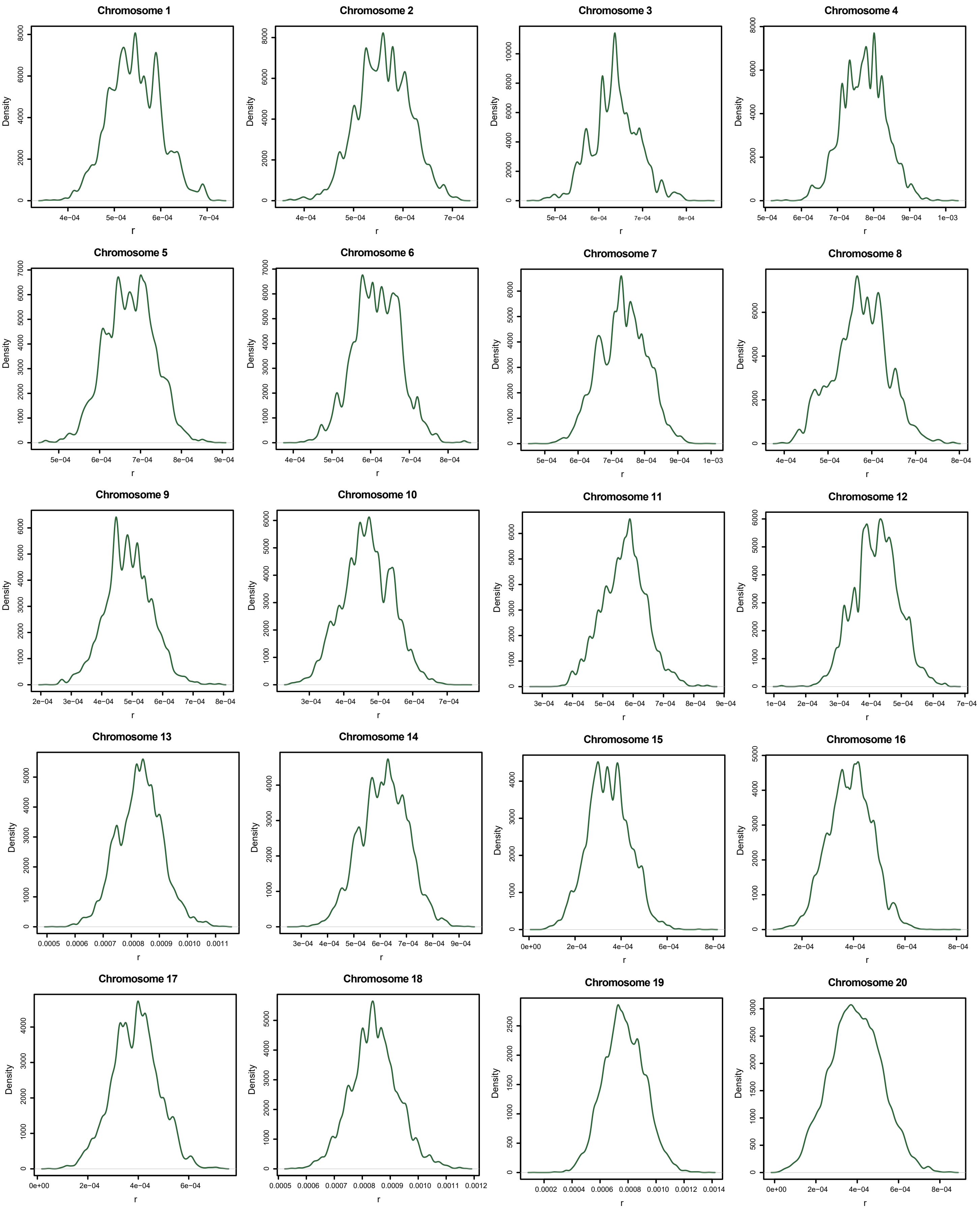

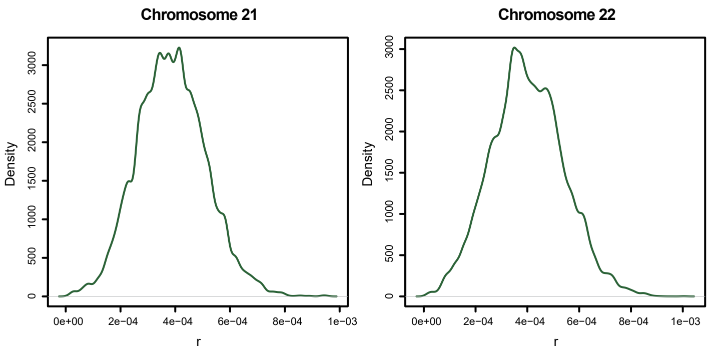

Supplementary Figure S10 Posterior probabilities for the contamination rate (r) estimated using the 2-pop method. 1KG CHB and JPT were used as anchor and contaminant populations, respectively.

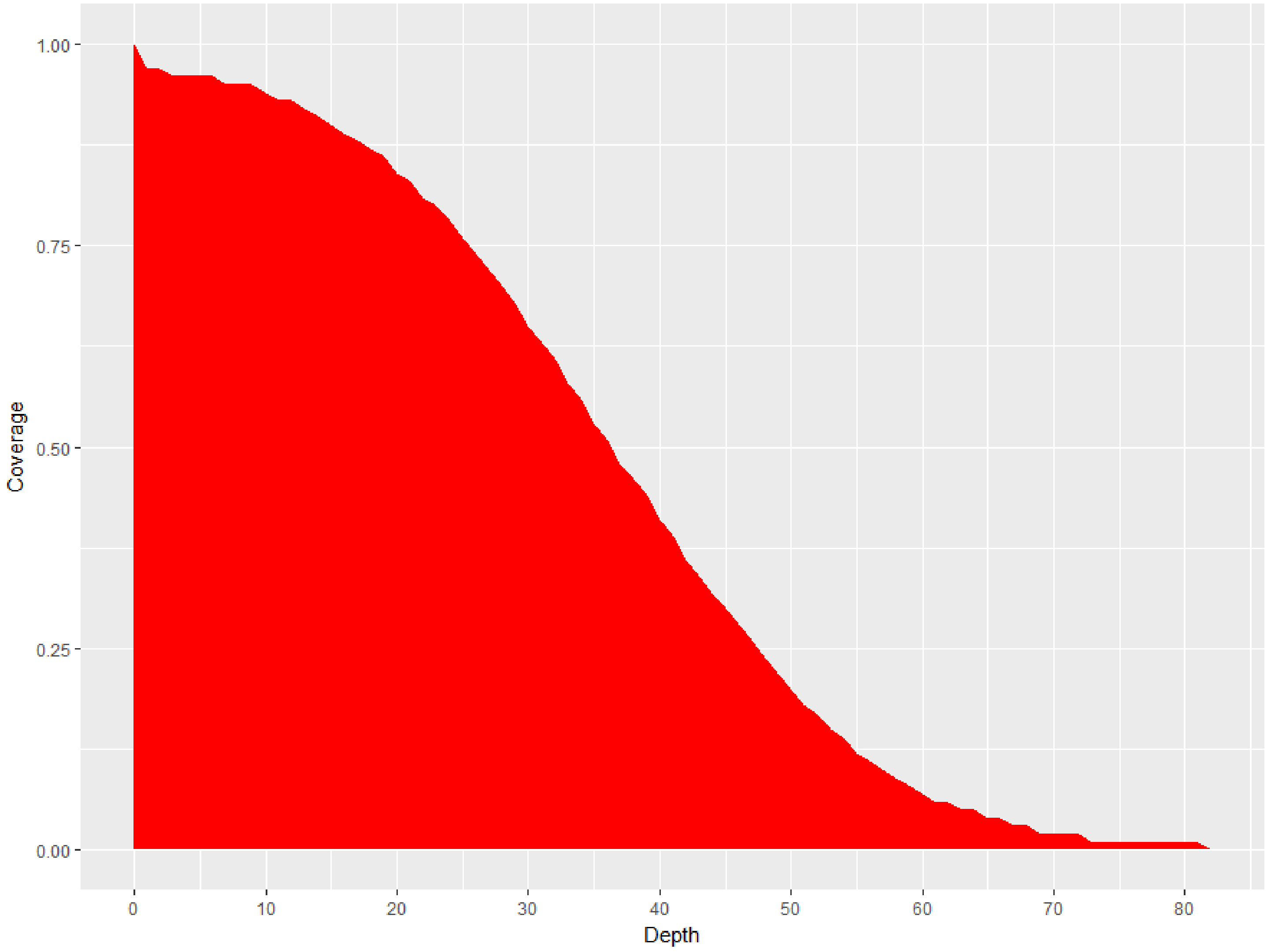

Supplementary Figure S11 Cumulataive distribution for the depth of coverage. The average depth of the NAT002 genome was 35×.

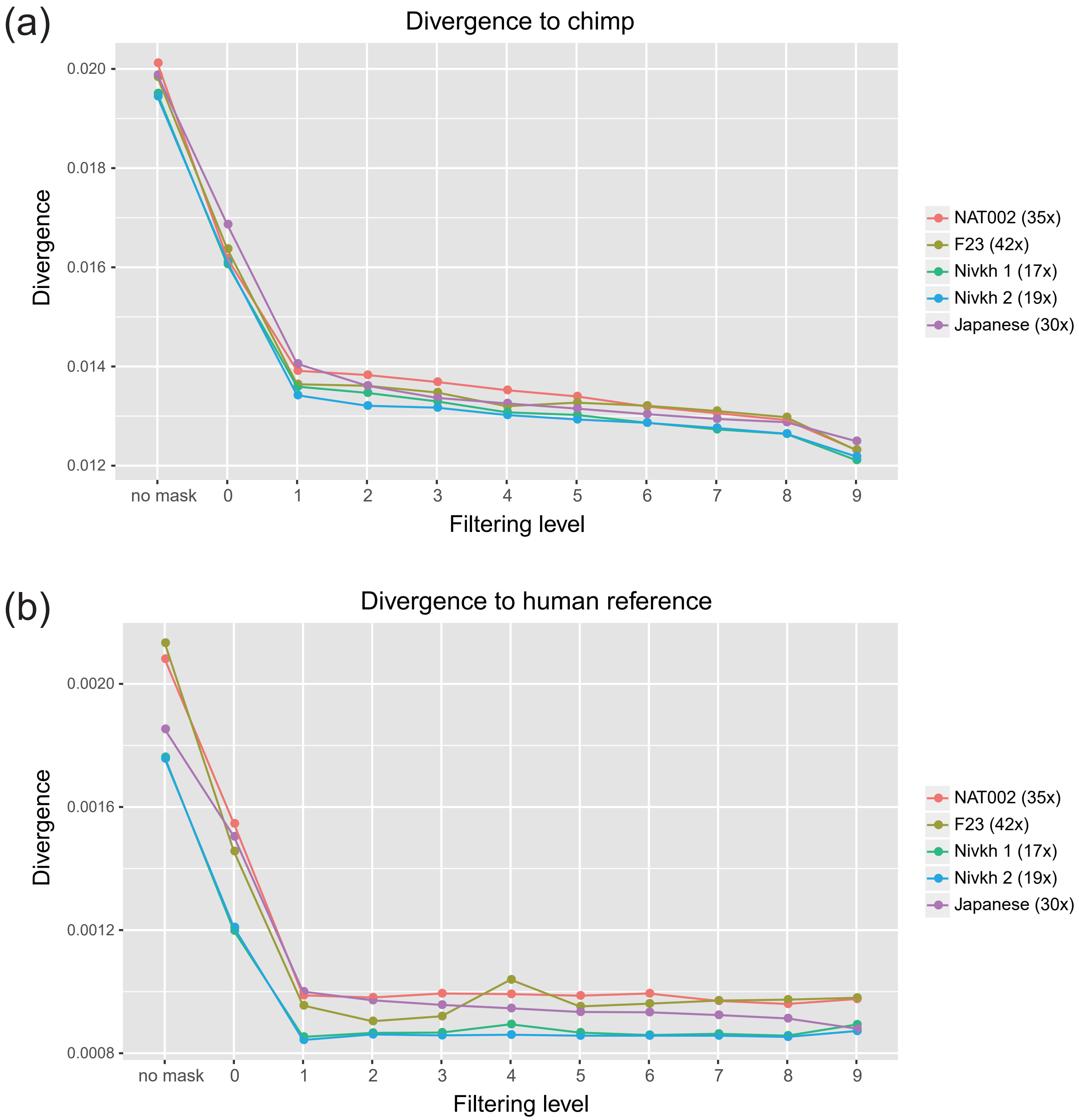

Supplementary Figure S12 Filtering results of NAT002, F23, two Nivkh individuals, and Japanese. **(a)** Divergence to chimpanzee and **(b)** divergence to human reference (hs37d5) for each filtering level. The divergences between the human reference and the Nivkh individulas were lower than the others, probably because of a reference bias caused by insuffcient depth.

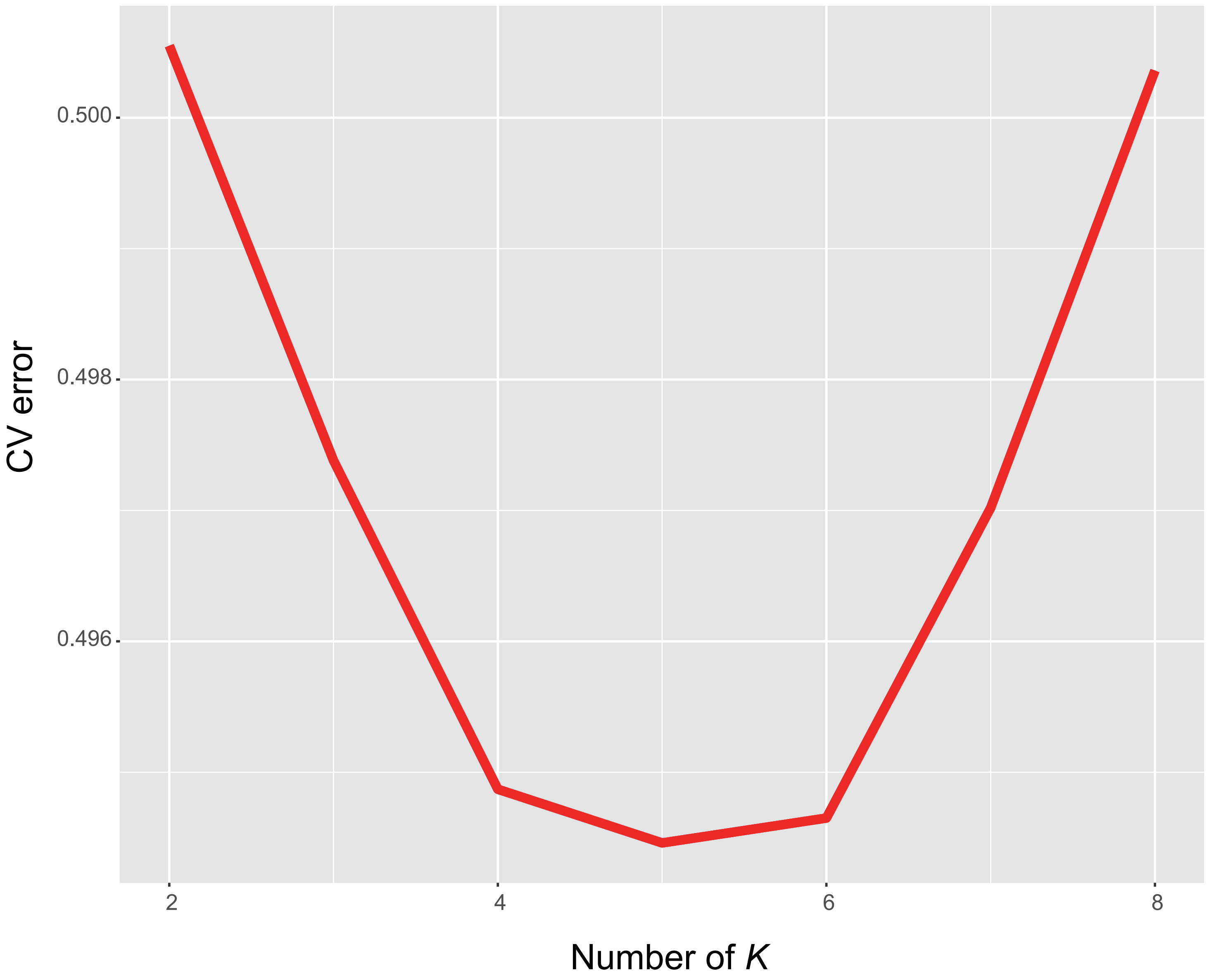

Supplementary Figure S13 The cross-validation errors when ADMIXTURE was run assuming *K*=2 to 10. The minimum CV error was observed when *K*=5.

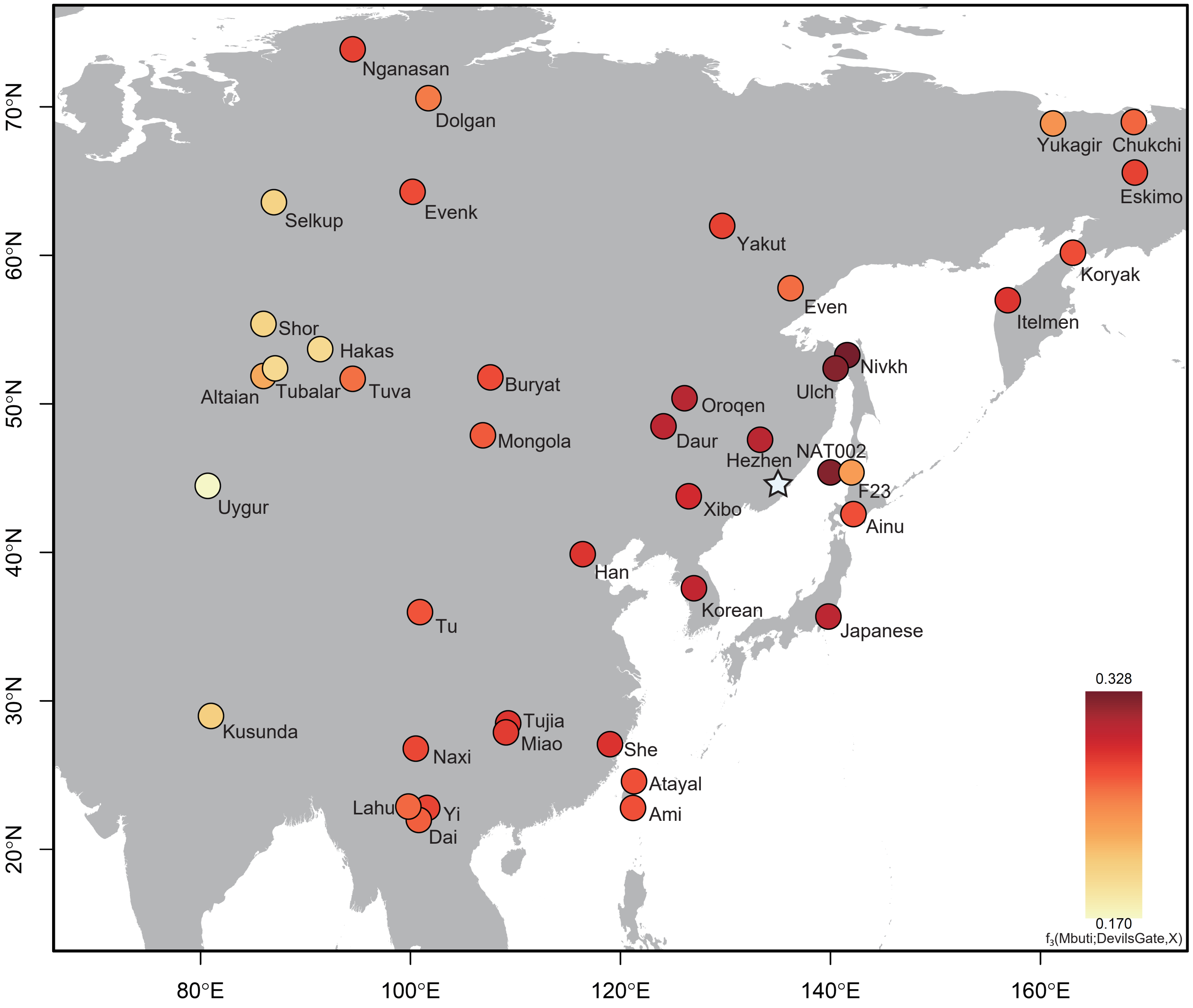

Supplementary Figure S14 The genetic affinities between the Devil's Gate individuals (Sikora et al. 2019) and neighboring populations. $f3$(Mbuti;DevilsGate,X) was computed. The star indicates the location of Devil's Gate Cave. NAT002 showed the second highest f_3 value following the Nivkh, among the tested populatrions. The other Amur populations also showed high affinities with Devil's Gate individuals. On the other hand, the Chukot-Kamchatka populations and F23 did not show very high affinities with Devil's Gate individuals.

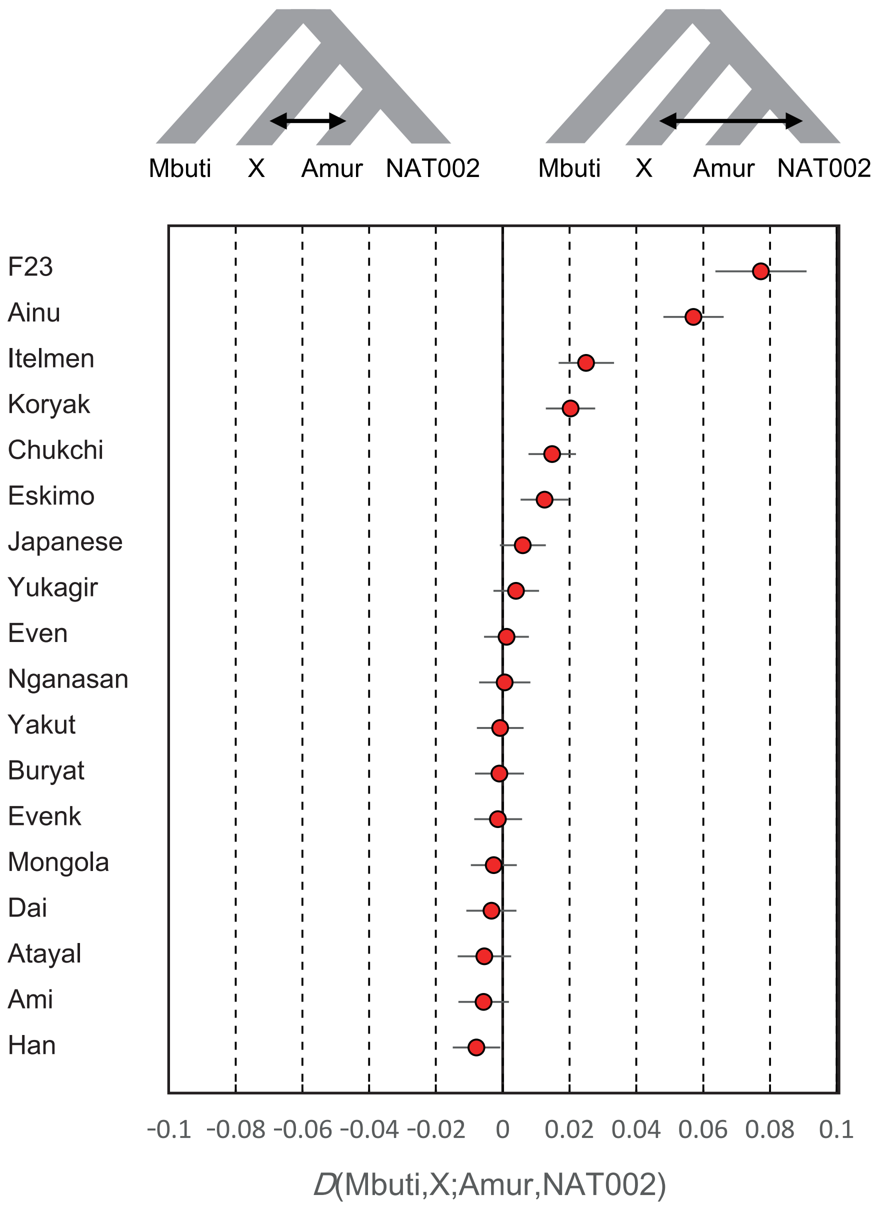

Supplementary Figure S15 The results of *D*(Mbuti,X;Amur,NAT002). Here, the Amur is a union of the Ulch, Oroqen, Hezhen, Daur, and Xibo. Error bars indicate two standard errors.

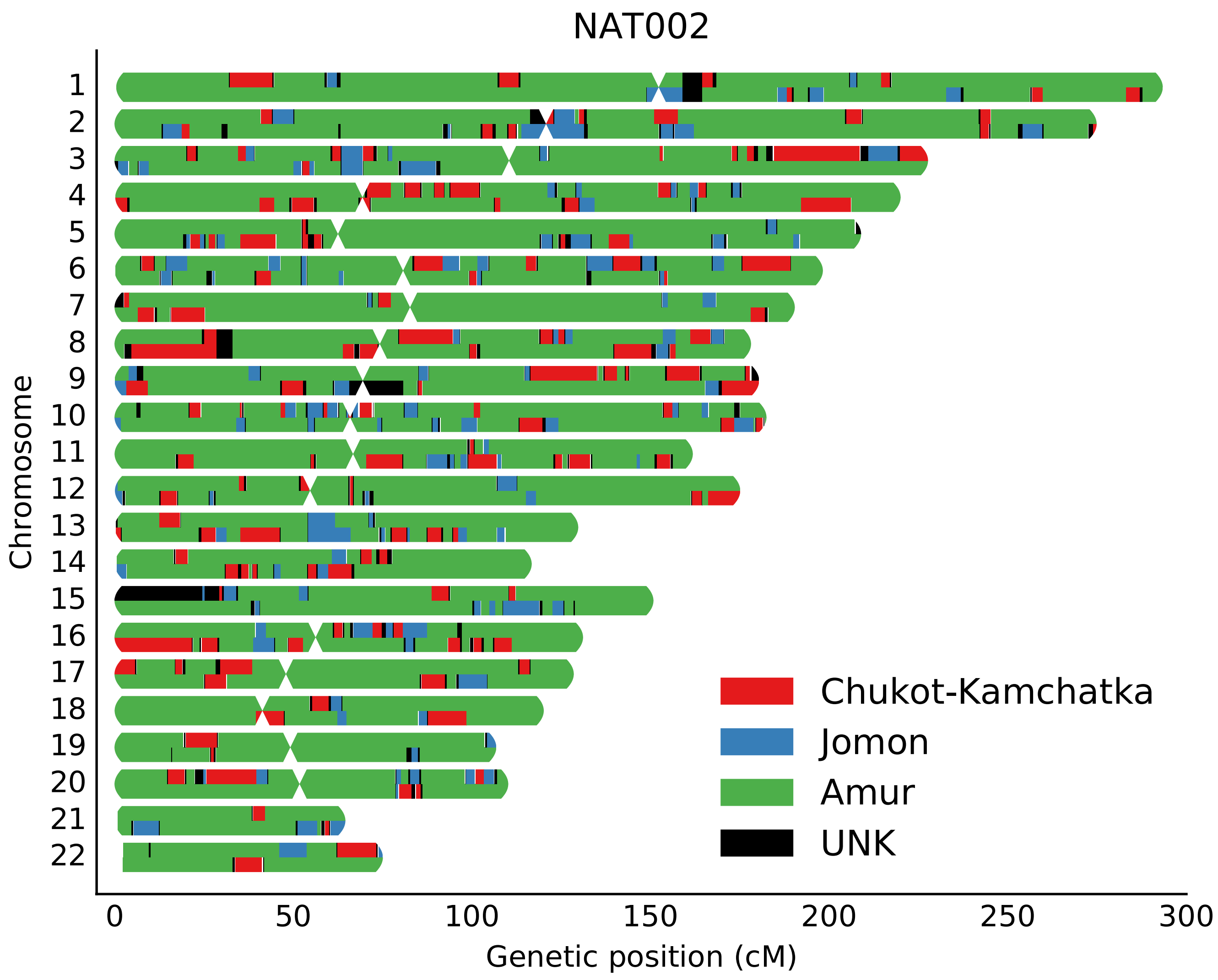

Supplementary Figure S16 Karyogram of NAT002 inferred by RFMix. Only F23 was used as a Jomon reference. UNK regions indicated posterior probabilities lower than 0.9.

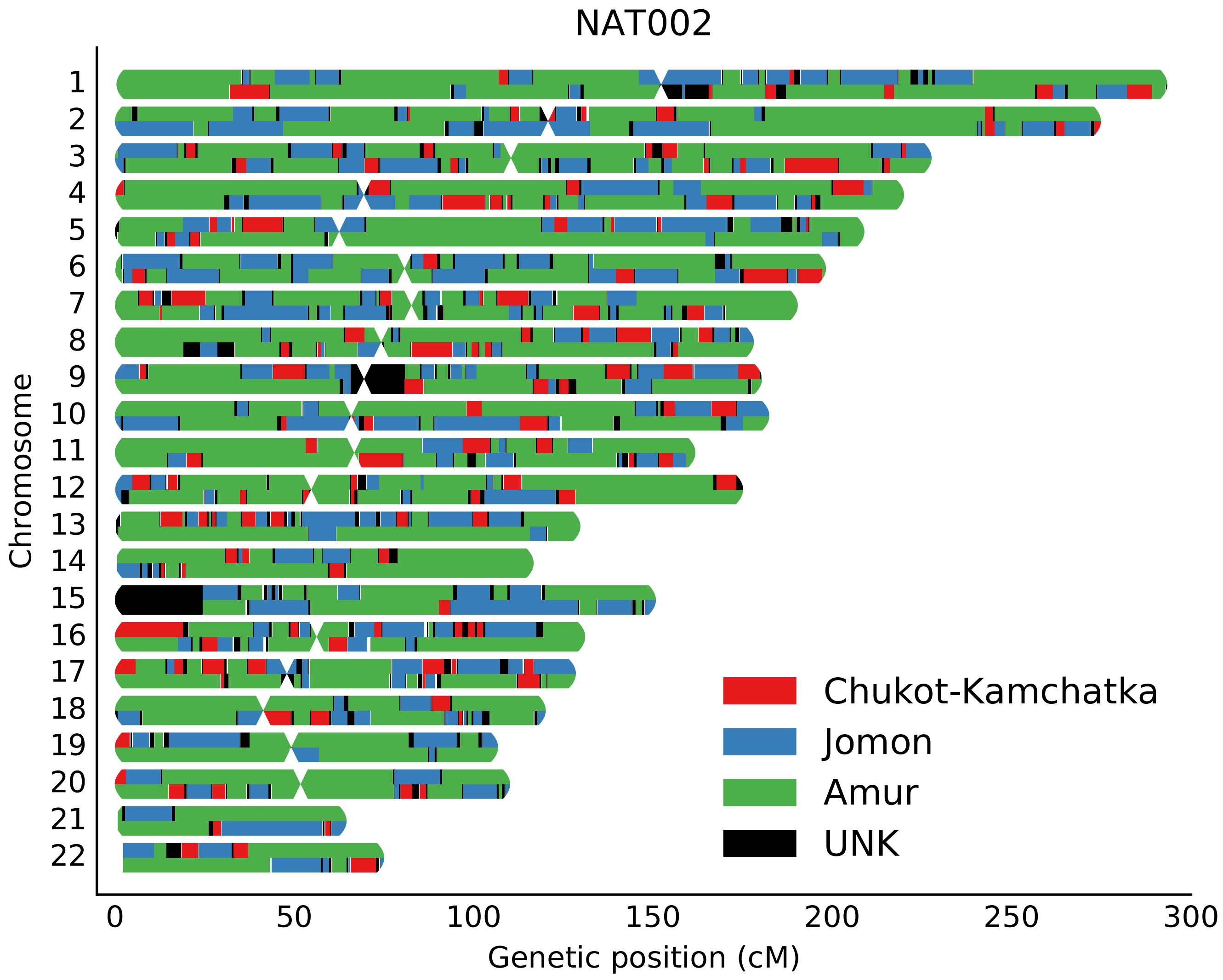

Supplementary Figure S17 Karyogram of NAT002 inferred by RFMix. F23 and modern Ainu individuals were used as Jomon references. UNK regions indicated posterior probabilities lower than 0.9.

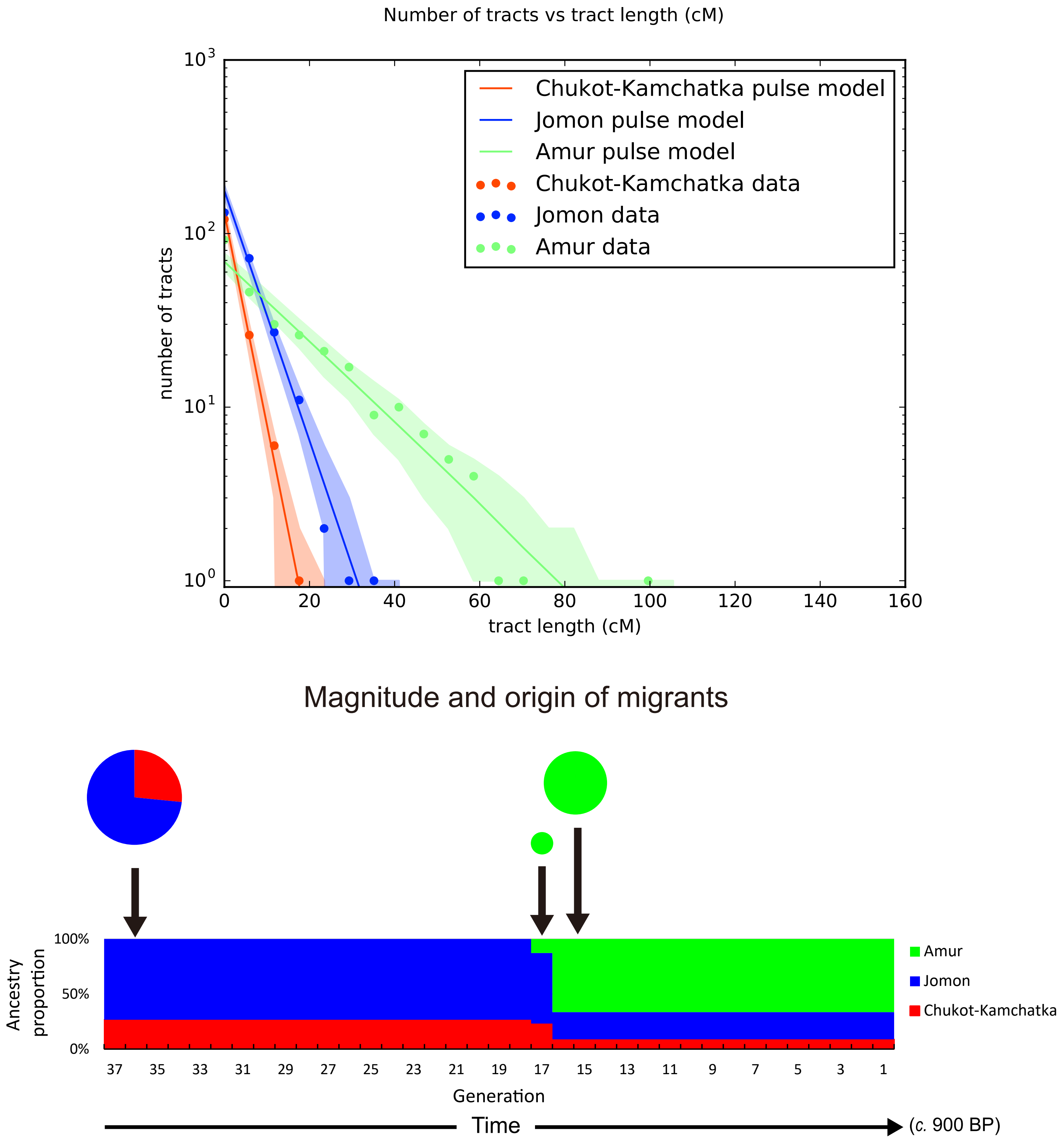

Supplementary Figure S18 Migration model inferred from the tract length distributions. Adding to F23, and 10 Ainu individuals were also used as a Jomon reference. The single pulse model of three populations was assumed. 0 generation corresponds to the ^{14}C age of NAT002 (*c.* 900 BP).

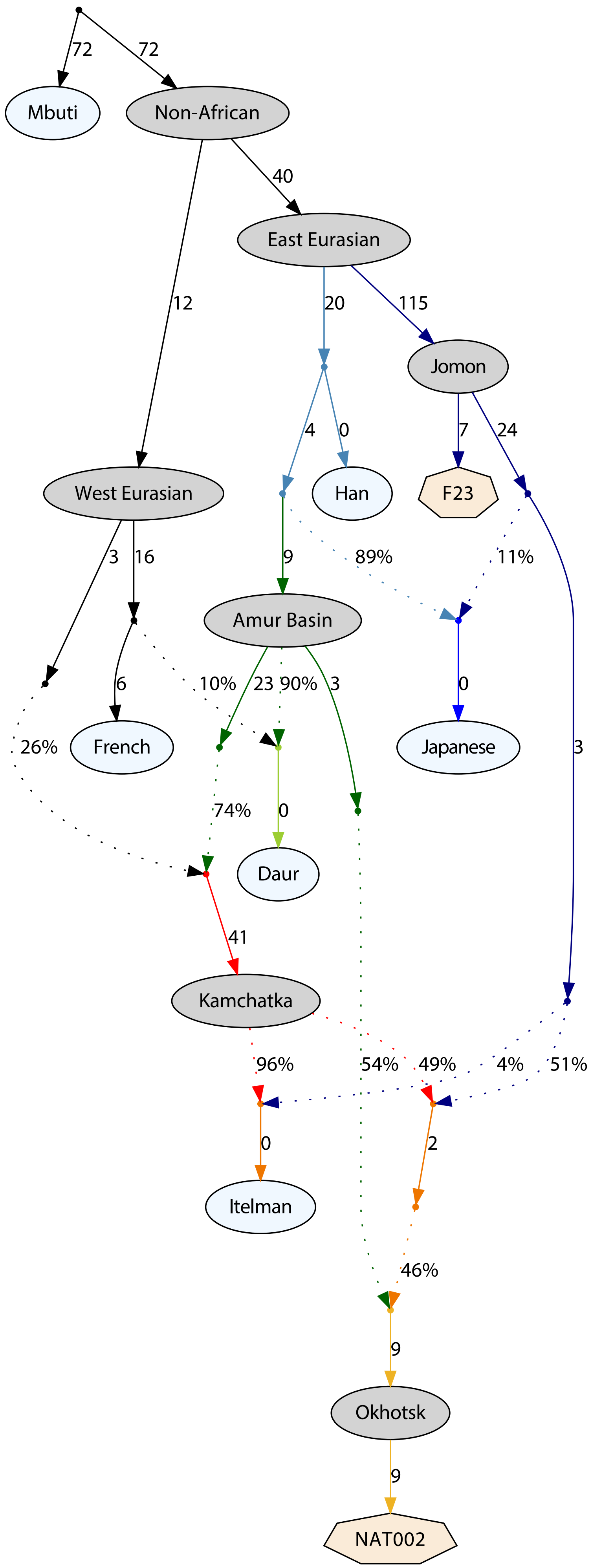

Supplementary Figure S19 Admixture graph involving NAT002, F23, and modern populations. NAT002 can be explained as an admixed individual among three lineages: the Amur, Kamchatka, and Jomon. The Z score for the worst f_4 statistic was -2.1.

(a)

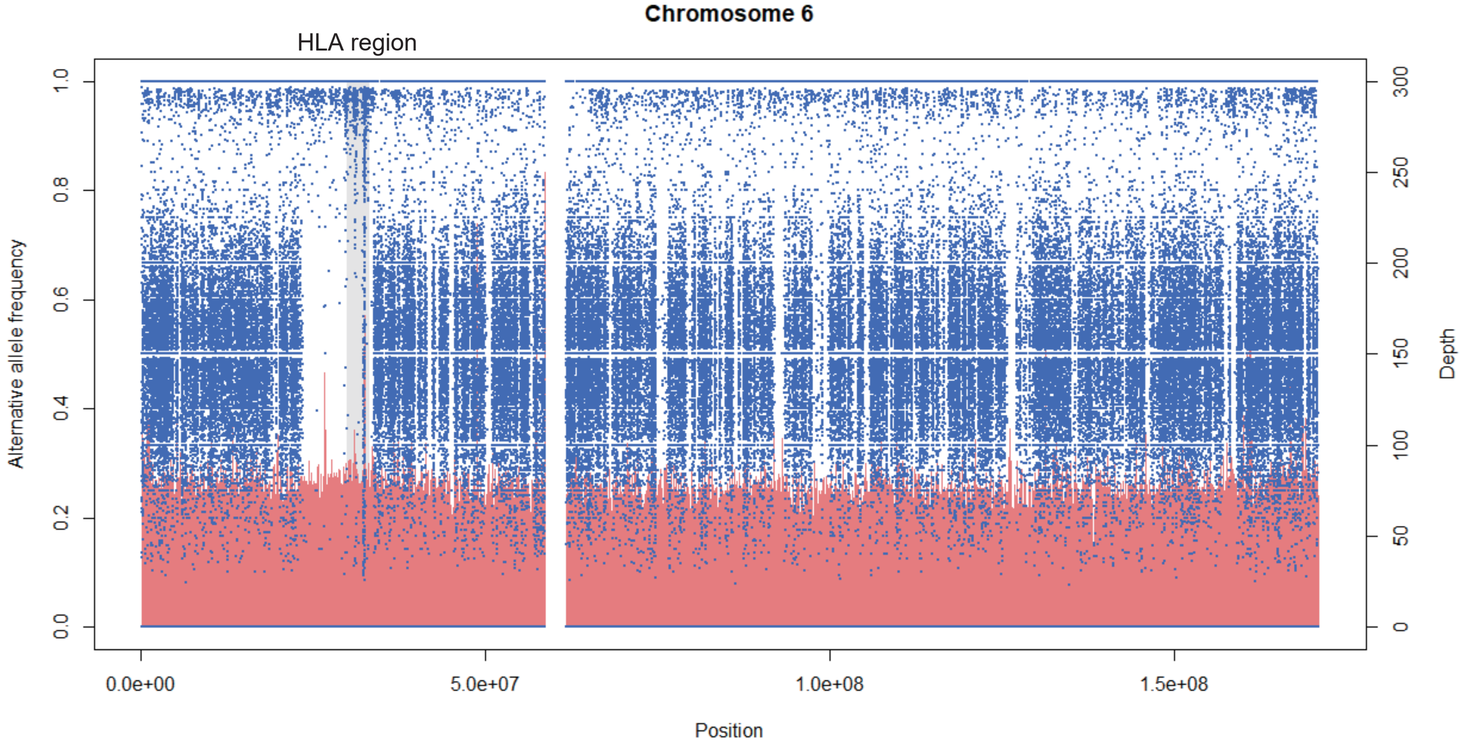

(b)

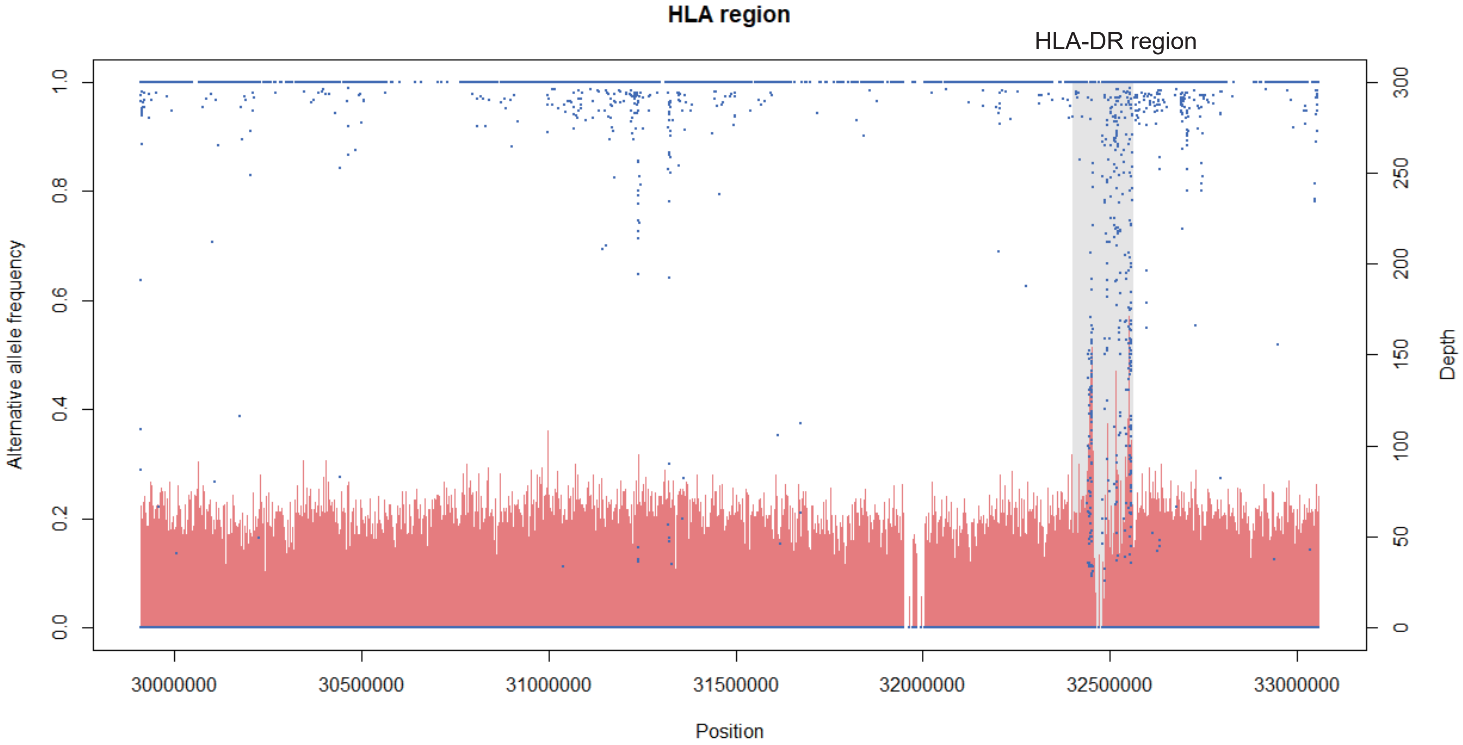

Supplementary Figure S20 Alternative allele frequencies and sequence depths at 1KG variant sites on **(a)** whole chromosome 6 and **(b)** HLA region in NAT002 sequence data. Only variant sites sequenced with depth ≥ 15x are plotted. Blue dot and red bar indicate alternative allele frequency and sequence depth at each site, respectively. **(a)** HLA region (including HLA Class I, Class II, and Class III) is shaded by grey color. **(b)** HLA-DR region (including *HLA-DRA, -DRB1*, *-DRB5*, and *-DRB6*) is shaded by grey color.

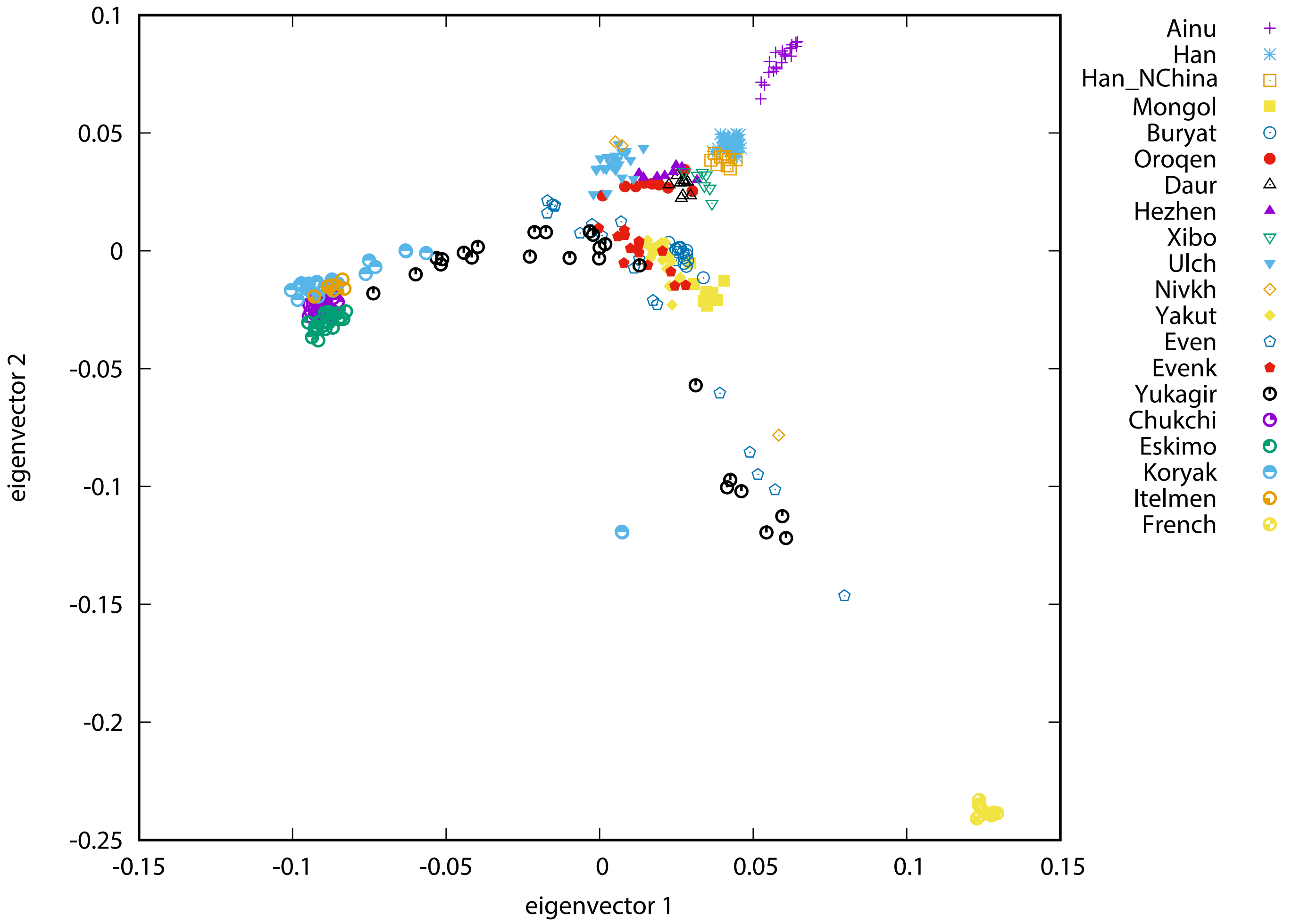

Supplementary Figure S21 PCA plot based on the East and Northeast Asian and French populations. The individuals plotted between the Asian and French clusters were regarded as recent admixed individuals and thus removed from subsequent analyses.
